# Moonlighting protein prediction using physico-chemical and evolutional properties via machine learning methods

**DOI:** 10.1186/s12859-021-04194-5

**Published:** 2021-05-24

**Authors:** Farshid Shirafkan, Sajjad Gharaghani, Karim Rahimian, Reza Hasan Sajedi, Javad Zahiri

**Affiliations:** 1 Laboratory of Bioinformatics and Drug Design, Institute of Biochemistry and Biophysics, University of Tehran, Tehran, Iran; 2 Bioinformatics and Computational Omics Lab (BioCOOL), Department of Biophysics, Faculty of Biological Sciences, Tarbiat Modares University, Tehran, Iran; 3 Department of Biochemistry, Faculty of Biological Sciences, Tarbiat Modares University, Tehran, Iran; 4 Department of Neuroscience, University of California San Diego, La Jolla, CA USA; 5 Department of Pediatrics, University of California San Diego, La Jolla, CA USA

**Keywords:** Moonlighting protein, Multitasking proteins, Physico-chemical properties, PSSM, Outlier, Random forest, SVM, bioinformatics

## Abstract

**Background:**

Moonlighting proteins (MPs) are a subclass of multifunctional proteins in which more than one independent or usually distinct function occurs in a single polypeptide chain. Identification of unknown cellular processes, understanding novel protein mechanisms, improving the prediction of protein functions, and gaining information about protein evolution are the main reasons to study MPs. They also play an important role in disease pathways and drug-target discovery. Since detecting MPs experimentally is quite a challenge, most of them are detected randomly. Therefore, introducing an appropriate computational approach to predict MPs seems reasonable.

**Results:**

In this study, we introduced a competent model for detecting moonlighting and non-MPs through extracted features from protein sequences. We attempted to set up a well-judged scheme for detecting outlier proteins. Consequently, 37 distinct feature vectors were utilized to study each protein’s impact on detecting MPs. Furthermore, 8 different classification methods were assessed to find the best performance. To detect outliers, each one of the classifications was executed 100 times by tenfold cross-validation on feature vectors; proteins which misclassified 90 times or more were grouped. This process was applied to every single feature vector and eventually the intersection of these groups was determined as the outlier proteins. The results of tenfold cross-validation on a dataset of 351 samples (containing 215 moonlighting and 136 non-moonlighting proteins) reveal that the SVM method on all feature vectors has the highest performance among all methods in this study and other available methods. Besides, the study of outliers showed that 57 of 351 proteins in the dataset could be an appropriate candidate for the outlier. Among the outlier proteins, there were non-MPs (such as P69797) that have been misclassified in 8 different classification methods with 16 different feature vectors. Because these proteins have been obtained by computational methods, the results of this study could reduce the likelihood of hypothesizing whether these proteins are non-moonlighting at all.

**Conclusions:**

MPs are difficult to be identified through experimentation. Using distinct feature vectors, our method enabled identification of novel moonlighting proteins. The study also pinpointed that a number of non-MPs are likely to be moonlighting.

**Supplementary Information:**

The online version contains supplementary material available at 10.1186/s12859-021-04194-5.

## Introduction

Recent cellular level research has produced interesting findings about protein functions. Protein function and its mechanism are present-day topics in biology [1]. One compelling reason beyond studying protein function is the latent importance of this vital macromolecule in the metabolism of organisms and pathogens. Although a considerable number of discovered proteins are multifunctional, most proteins are unifunctional. Moonlighting proteins (MPs) comprise a subset of multifunctional proteins in which one polypeptide chain exhibits more than one biochemical or biophysical function [2].

To be more precise, the word moonlight can be applied to proteins with at least two different unrelated functions providing this multifunctionality is not as a result of gene fusion, multiple domains, multiple splice variants, proteolytic fragments, families of homologous, or pleitropic effect [3]. Independence of functions is another important feature of the MPs; the inactivation of one function does not affect other protein functions [4].

The first example of MPs is reported in the late 1980s by Piatigorsky and Wistow [5]. They noticed that crystallin, a structural protein in the eye lens, has an enzymatic role as well. Hitherto these proteins have been discovered in mammals, yeast, worms, bacteria, plants, viruses, archaea, and many other organisms. To record the data related to these proteins several online databases are established. MoonProt [6] and MultitaskProtDB-II [7] and MoonDB [8] have reported 400 and 694 and 238 proteins respectively, in their last update. MPs contain various sub-types: (1) different sites for different functions in the same domain (2) different sites for different domains in different domains (3) implementing the same residue for different functions (4) implementing different residues of the same site for different functions (5) implementing structural composition or different folding for different functions [9]. Although there have been several studies on MPs in recent decades, a great deal about these proteins (such as the number of these proteins) still remains unknown. Detection of protein functions, how to target a function without affecting other functions, and discovering the expression patterns changes to find a novel function are among the major questions in biology, which deserve convincing scientific answers [10]. In addition to the mentioned matters, detecting unknown cellular processes, identifying new protein mechanisms, improving protein function prediction, a significant role in disease pathways, obtaining information on protein evolution, and drug discovery are the reasons that make MP studies more appealing. According to previous studies, 78% of MPs are involved in human disease pathways and 48% of MPs are the targets of active medicines [11]. For example, phosphoglucose isomerase is an enzyme in glycolysis and also is a cytokine (autocrine motility factor), which has a significant role in breast cancer metastasis [12]. Several other research findings are provided in [13]. The reasons mentioned above on the one side and the challenging laboratory and experimentally methods in detecting these proteins on the other have made computational methods so remarkable. To date, several computational methods have been used to detect moonlighting proteins. Since moonlighting proteins, tend to interact with other proteins with different functions or in different pathways, they can be detected by protein–protein interaction (PPI) [14]. Hernandez et al. implemented sequence similarity to other protein families with\different functions to detect MPs [15].

Chapple et al. used a protein–protein interaction network to extract features that enable them to identify extreme multifunctional proteins [16]. According to Chapple et al., these types of proteins belong to several functional modules that are engaged in different functions with MPs as one of their subclasses. In their study, they detected the overlapping cluster of a PPI network. These clusters contained highly interconnected proteins that tended to get involved in the identical cellular process. In the next phase, clusters were annotated by the common function of most of the clusters’ proteins. Proteins that were found in the intersection of the clusters were then selected as the candidates. The candidate proteins had more than one function and their first and second functions were not identical. They observed that the number, degree, and the relationship of domains with the disease in candidate proteins were more than the ones in proteins that were in the intersection of clusters but had not been selected due to the identical first and second functions. Also, the average degree in the candidate proteins is higher than the hubs but candidates are less disordered than the hubs.

Jain et al. developed a new method by text mining to detect moonlighting protein using various information sources [1]. In their method, moonlighting proteins were detected by analyzing database entries, literature, and big data omics utilizing the DextMP algorithm. Their research was applied to the genome proteins of Arabidopsis thaliana, Caenorhabditis elegans, and Drosophila melanogaster. In another study by Khan et al. [17], the functional features of MPs were identified by using a computational framework from various proteomics aspects. They created a model for prediction of moonlighting protein based on gene ontology (GO), PPI, gene expression, phylogenetic profiles, genetic interactions, network-based graph properties, and the number and length of intrinsically disordered regions. The prediction accuracy of this method by applying the random forest classification algorithm was 72%. In a study by Khan et al. (2016), Go annotation was used to predict MPs and was able to identify these proteins with an accuracy of 0.98 [18]. Although this method was very accurate, the lack of Go annotation for all available proteins was one of its main constraints. The use of information in amino acid sequences is still one of the main methods of identifying moonlighting proteins that are currently being researched extensively. In the present study, we aimed to investigate the effect of 37 different feature vectors extractable from amino acid sequences in distinguishing MPs from non-MP and introduce the best feature vector. To do this, 8 famous classification models that with various applications in bioinformatics were used.

## Material and methods

### Dataset

A dataset of 351 proteins was utilized that contained 136 non-moonlighting and 215 moonlighting proteins. This dataset contained proteins derived from different organisms. Table 1 presents the number of proteins based on each organism for each class. To collect moonlighting proteins, the MoonProt database (http://www.moonlightingproteins.org/) and for non-moonlighting proteins, Khan et al. [17] method based on function annotation were utilized. To date, the moonlight database contains 400 MPs and the set of proteins that were introduced by the khan method are 150 samples. Since data redundancy can lead to bias, CD-hit was utilized to remove the redundant or similar protein. The sequence identity cut-off was considered 40. Finally, a set of 351 proteins was obtained. List of moonlighting and non-moonlighting proteins sequence available in Additional file 1 and Additional file 2.

**Table 1:** The number of moonlighting and non-moonlighting proteins. (moonlight exist in different organism)

Organism	Moonlight	Non-Moonlight
Mus Musclus	11	39
Human	57	48
*E. coli*	24	16
Yeast	23	33
Rat	5	0
Drome	8	0
Arath	5	0
Other	82	0
Total	215	136

### Feature extraction

Thirty-seven feature vectors used in this study are presented in Table 2. The name of each set of features, length of the feature vector, and a brief definition are described in Table 2. All feature vectors from 1 to 36 were extracted by the ftrCOOL library [19]. The IF set of features, which is provided in Table 2 under number 37, consists of several features each of which extracted with an appropriate tool. These features are include length, molecular mass, isoelectric point, charge, hydrophobicity, aliphatic index, instability index, GC-content, hydrogen binding, number of hydrogen bond in alpha-helix (h-Alpha Helix), number of hydrogen bond in beta-sheet (h-BetaSheet), Kidera factor features, MS-WHIM score, post-translational modification, disorder, Amino Acid Composition, Pseudo Amino Acid Composition(PseAAC), and position-specific scoring matrix (PSSM). The PSSM set of features was extracted by the bioinformatics tool POSSUM [20]. For more details about each feature vector see Additional file 3.

**Table 2:** Feature vectors extracted for protein sequences

Row#	Feature vector	Description	Vector length #
1	AAKpart composition	Grouped amino acid K part composition	60
2	AAutoCor	Amino acid autocorrelation-autocovariance	456
3	CkSAApair	Composition of k-spaced amino acids pairs	400
4	CkSGAApair	Composition of k-spaced grouped amino acids pairs	64
5	CTD	Composition transition distribution	147
6	CTDC	Composition transition distribution composition	21
7	CTDD	CTD distribution	105
8	DDE	Dipeptide deviation from expected mean value	400
9	ExpectedValueAA	Expected value for each amino acid	400
10	ExpectedValueGAA	Expected value for grouped amino acid	512
11	ExpectedValueGKmerAA	Expected value for grouped K-mer amino acid	64
12	ExpectedValueKmerAA	Expected value for K-mer amino acid	400
13	GrpDDE	Group dipeptide deviation from expected mean	64
14	SOCNumber	Sequence order coupling number	60
15	kAAComposition	k Amino acid composition	8000
16	kGAAComposition	k Grouped amino acid composition	512
17	PseKRAAC-T1	Pseudo K-tuple reduced amino acid composition Type-1	16
18	PseKRAAC-T10	Pseudo K-tuple reduced amino acid composition Type-10	625
19	PseKRAAC-T11	Pseudo K-tuple reduced amino acid composition Type-11	625
20	PseKRAAC-T12	Pseudo K-tuple reduced amino acid composition Type-12	625
21	PseKRAAC-T13	Pseudo K-tuple reduced amino acid composition Type-13	256
22	PseKRAAC-T14	Pseudo K-tuple reduced amino acid composition Type-14	16
23	PseKRAAC-T15	Pseudo K-tuple reduced amino acid composition Type-15	16
24	PseKRAAC-T16	Pseudo K-tuple reduced amino acid composition Type-16	16
25	PseKRAAC-T3A	Pseudo K-tuple reduced amino acid composition Type-3A	16
26	PseKRAAC-T3B	Pseudo K-tuple reduced amino acid composition Type-3B	16
27	PseKRAAC-T4	Pseudo K-tuple reduced amino acid composition Type-4	625
28	PseKRAAC-T5	Pseudo K-tuple reduced amino acid composition Type-4	256
29	PseKRAAC-T6A	Pseudo K-tuple reduced amino acid composition Type-6A	625
30	PseKRAAC-T6B	Pseudo K-tuple reduced amino acid composition Type-6B	625
31	PseKRAAC-T7	Pseudo K-tuple reduced amino acid composition Type-7	625
32	PseKRAAC-T8	Pseudo K-tuple reduced amino acid composition Type-8	625
33	PseKRAAC-T9	Pseudo K-tuple reduced amino acid composition Type-9	625
34	QSOrder	Quasi sequence order	50
35	SAAC	Splitted amino acid composition	60
36	SGAAC	Splitted group amino acid composition	24
37	IF	Interest feature	106

### Machine learning methods

The classification methods used in this study were Support Vector Machine (SVM), K nearest neighbor (KNN), Na¨ıve Bayes (NB), Decision Tree (DT), Random Forest (RF), Multi-Layer perceptron (MLP), Ada Boost (ADA), Logistic Regression (LR). SVM is one of the most applicable methods of machine learning that utilizes an optimized hyperplane to distinguish classes [21]. One of the advantages of this method is unerring accuracy and high performance. SVM is used for hot spot detection in proteins. KNN is one of the simplest algorithms of machine learning [22]. In KNN, the distance of an object to the k nearest neighbors is calculated, and then the object adapts the label that has the most numbers between k nearest objects. Prediction of the hot spot in proteins and PPI are the applications of this method [23, 24].

NB classification method is based on Bayes’ theorem and independence assumptions between the data. This assumption can significantly reduce the complexity of the classification. Simplicity and low computational costs are the advantages of this method while the independence assumption and normalization of data distribution could have a negative impact on the accuracy and precision of the algorithm. Several successful applications of this method have been reported for PPI [25, 26].

DT is one of the most useful classification methods that can visualize the relation between classes and feature vector [27]. Each leaf of the decision tree represents a class. Branches perform as ways to classes based on the contents of the feature vectors. Although this method is simple, it can increase the classification error. This method is successful in PPI prediction [28].

RF is a collection of decision trees. Every decision tree is trained by a subset of features. The selection of this subset is done randomly. Ultimately, to calculate the predicted value, the majority of votes are used. This method has been successful in predicting PPI that was presented by Akbaripour-Elahabad et al. [29].

MLP artificial neural networks are made based on human contemplation that can process simultaneously [30]. Multilayer neural network is a type of artificial neural network that consists of at least three layers: input, hidden, and output. Each layer contains several nodes (neurons) that exhibit a specific output in the network. Edges connect the nodes and each edge contains a value called weight. The edges transfer output of a neuron to another. The last layer is the output layer and the result could be extracted from this layer. This network demonstrates good results in human virus PPI prediction [31].

ADA is one of the ensemble methods that the compositions of classifiers are used for better and more accurate predictions. In this method, weaker classifiers called week learners are utilized. Each week learner creates an output (a class) for each sample. Then the linear sum of these week learners is selected so that the classifier error is minimized. LR, despite its name, is a statistical model for classification problems rather than regression. Although many more complex extensions exist, in the basic form it uses a logistic function to model dichotomous classification problems. The logistic function, also called the sigmoid function was developed to describe feature of population grow in ecology, rising quickly at the carrying capacity of environment. In this method, instead of fitting a straight line or hyperplane, the logistic regression model uses the logistic function to squeeze the output of a linear between 0 and 1. LR has been successful in prediction protein function from protein–protein interaction data [32].

### Run Models

To run the model, we required to extract each of the feature vectors presented in Table 2. To do this, the FASTA file containing the moonlighting and non-moonlighting protein sequences was given as input to the ftrCOOL package in R. Each of the extracted feature vectors was then stored in a file. Each of the introduced feature vectors had parameters to set. We used the default parameters in the ftrCOOL package. For instance, to calculate the SAAC feature vector, the N-terminal and C-terminal parameters were set to 5. The default parameters for each feature vector can be observed in [19]. After extraction of feature vectors, 20% of the proteins were randomly selected and reserved as test data. The remaining 80% was used to learn each of the classification models through 100*tenfold cross-validation method. The proteins selected in each fold were considered for all methods as per each feature vector, so a bias-free comparison can be obtained. A very popular library, the scikit-learn library, was used to run classifier models.

**Fig. 1:**
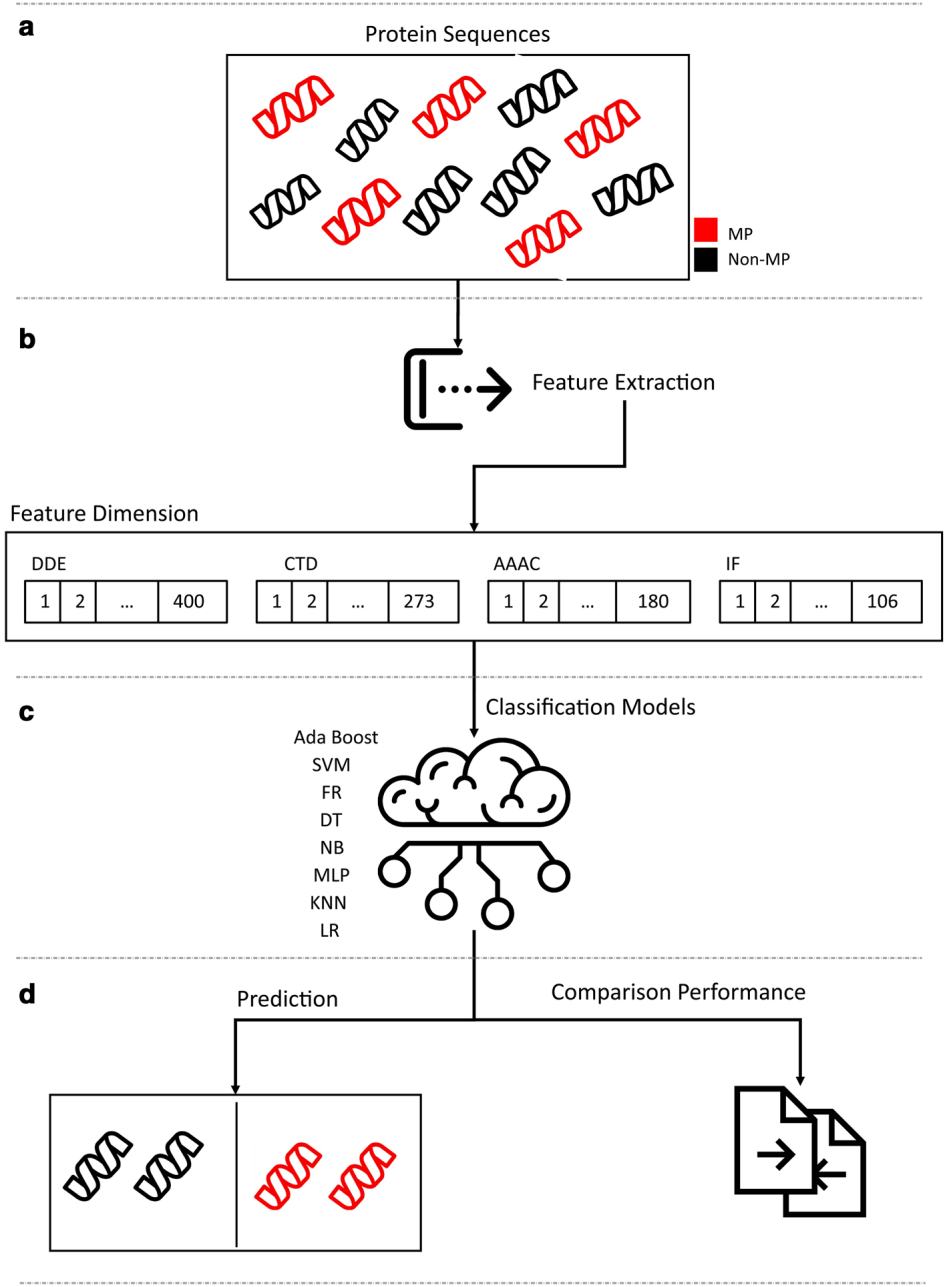
Schematic view of Pipeline for moonlighting proteins detections. **A** Collecting proteins. **B** Extracting features. **C** Training models (MLP, KNN, LR, Ada Boost, SVM, RF, DT, NB). **D** Performance evaluation

Each model has its set of parameters to get the best results and they ought to be adjusted. The best value of k in KNN was 5 and the Euclidean distance function was set. The kernel function in the SVM method was set equal to RBF and marginal parameter C was set to 1. In the decision tree, the Gini criteria was used, and max-depth, and min-sample was set to 3 and 5, respectively. The number of weak learners, in the Ada boost method, considered 200, and the number of decision trees in RF method was set equal to 50. Ultimately, to perform and execute MLP, two layers of 20 and 3 neurons in the hidden layer with the maximum iteration of 150 were used. Out of all available activation functions for this method, sgd exhibited better results. Figure 1 shows the implementation of classification models. First, desirable features (Table 2) were extracted from protein sequences (MPs and non-MPs). Each of the features in Table 2 was saved in separate vectors with different dimensions. Then each of the feature vectors was used for classification model training. The trained models were compared to one another and the best feature vector and also the best model were selected. Finally, the best model was used for protein classification.

### Performance evaluation

Sixteen feature vectors and 8 classification methods were studied in this research that produced a total of 128 different results. To achieve the best result, tenfold cross-validation was used and the result assessment was done using F-measure, Precision, Recall, Accuracy (acc), and MCC.

1 $$acc = \left( {TP + TN} \right)/\left( {TP + FP + TN + FN} \right)$$

2 $$precision = \left( {TP} \right)/\left( {TP + FP} \right)$$

3 $$recall = TP/\left( {TP + FN} \right)$$

4 $$F - Measure = 2 \times \left( {precision \times recall} \right)/\left( {precision + recall} \right)$$

5 $$MCC = \left( {TP \times TN - FP \times FN} \right)/{ }\surd \left( {TP + FP} \right)\left( {TP + FN} \right)\left( {TN + FP} \right)\left( {TN + FN} \right){ }$$

In these equations, TP represents the number of true positives, FP, TN, and FN show the number of false positives, true negatives, and false negatives, respectively. For further information refer to [33]. The area under the curve of ROC (AUC) was utilized as well.

## Results and discussion

### Results of model performance

Because the detection of a moonlighting protein is carried out randomly, the use of computational methods and classification can be very helpful in determining whether a protein is monolithic. Regarding the idea of using outliers, it can be said that outlier samples can significantly reduce the performance of classifier models, and because non-MP proteins do not have laboratory approval, they are prone to a lot of error. This can lead to outdated specimens. This is not the case with MPs because they have been confirmed by experimental methods; nevertheless, they may contain proteins that are different from other proteins, and this can affect the efficiency of the classification methods. In the present study, we attempted to identify proteins that reduce the accuracy of classification models.

**Fig. 2:**
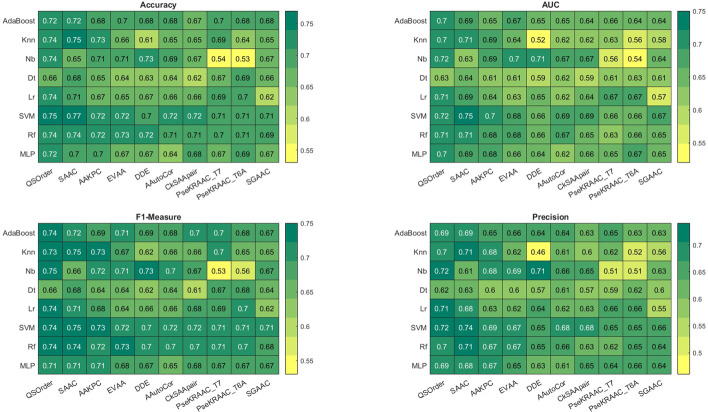
Models cross-validation performance. Using the heat map to compare performance (Accuracy, Recall, Precision, F-Measure, and AUC) of models. Red and green colors indicate worse and better results, respectively

**Fig. 3:**
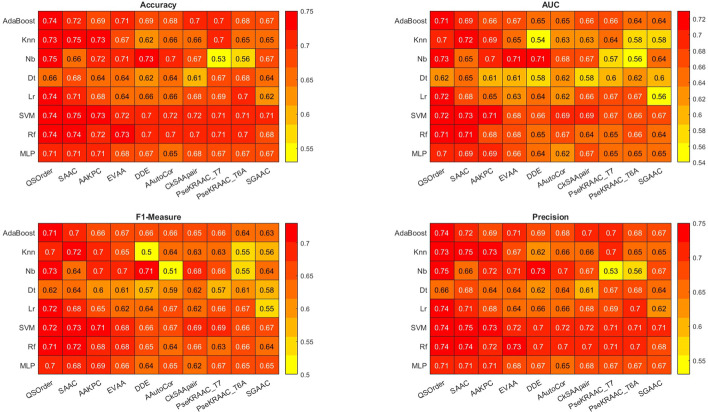
Models test performance. Using the heat map to compare performance (Accuracy, Precision, F-Measure, and AUC) of models. Red and green colors indicate worse and better results, respectively

To obtain the results, 20% of the proteins were set aside as test data and the remaining 80% of the proteins were used by tenfold cross validation method to learn the classification models. To increase the level of reliability of the results (selecting 20% test data and 80% training data, randomly), each feature vector was tested100 times and each time the values of accuracy, precision, recall, MCC, F-Measure were calculated, finally the average was reported as the final result. For convenience, we show this method as 100 times tenfold cross validation (100*tenfold CV). The proteins selected for the test and training sets in each iteration are assumed to be the same for all feature vectors and classification models. This issue was also observed for each of the folds in the tenfold CV method so that the obtained results are comparable away from any bias. To run this program, a 6-core computer with 16 GB of RAM was used and lasted about 18 h. 100*tenfold CV was performed separately on 37 feature vectors and from among them, 10 sets of vectors that had higher performance than the others were selected through 100*tenfold CV. Figure 2 shows the results. The results for the other feature vectors are given in Additional file 4. Observing the results, it is clear that the SVM model using the SAAC feature set has an accuracy of 0.77%, which has the highest accuracy in the whole feature set. Also, QSorder and SAAC feature sets perform better than other features in distinguishing MPs from non-MP, so that the average accuracy for all classification models in the QSorder feature set is 0.72 and for the SAAC feature set is 0.71%, which have the first and second highest percentages, respectively. The results obtained on the test proteins also confirm this issue. Figure 3 shows the results of implementing classification models on 10 superior feature vectors. As can be seen, the SVM method using the SAAC feature set, the NB method using the QSorder feature set and the KNN method using the SAAC feature set reveal an accuracy of 75%, which is the highest accuracy in the test data set. Also, RF, SVM, Adaboost, LR methods using QSorder feature set and RF using SAAC feature set have 74% accuracy. This indicates that the two feature sets QSorder and SAAC can better distinguish MPs from non-MP proteins than the other feature vectors investigated in this study. The results show that the SAAC feature vector based on tenfold CV criterion has an accuracy of 0.77 and based on test data has an accuracy of 0.75%, which is higher than method [17] and equivalent to method [18]. However, for comparison without bias, the set of proteins collected in the study [18] was used and the SAAC specificity was calculated for them. Table 3 shows the performance result of tenfold cross validation for this data. As can be seen, the SVM method using the SAAC feature vector has an accuracy of 0.817, which is higher than the mpfit operation in [18]. This suggests that the SAAC property could be a good candidate for distinguishing MPs from non-MP proteins. And SVM classification using the SAAC feature set can outperform similar methods.

**Table 3:** MpFit dataset performance

Performance measure	ADA	KNN	NB	DT	LR	SVM	RF	MLP
AUC	0.739	0.765	0.654	0.685	0.748	0.806	0.784	0.780
ACC	0.752	0.793	0.712	0.697	0.765	0.817	0.789	0.796
F1	0.739	0.770	0.653	0.680	0.749	0.802	0.796	0.781
Precision	0.752	0.793	0.712	0.697	0.765	0.813	0.779	0.796

### Outlier detection

**Fig. 4:**
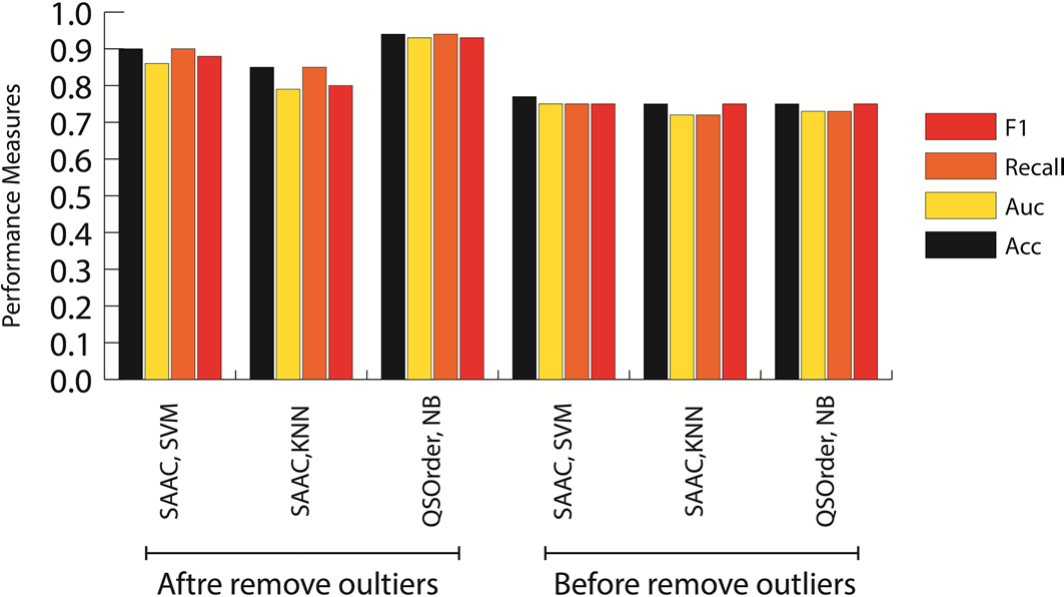
Cross validation performance after remove outliers

Outlier samples can be error-bound (for example, data entry point, measurement error, experimental error, sampling errors) or have no error, in which case they are called natural outlier. In other words, natural outliers are actually samples that do not make any errors, but their distance from the rest of the samples is considerably large [34]. There are different ways to identify outlier proteins. This study used counts of proteins that were misclassified. In this method, a category was trained using a feature set through tenfold cross validation. The set of proteins was then divided into tenfold, so that nine-fold was considered as a train and one part as validation, with the model data of the trained model and with the data set validation. The efficiency of the model was checked. This was repeated 10 times and each time one of the folds was considered as validation data, finally the average of every 10 times was reported as the final result. Obviously, each time a tenfold cross validation was performed, each protein must have been included in the validation set and only once. To identify outlier proteins, the above method was performed 100 times and each time the proteins that were incorrectly classified in the validation set were counted. If a protein was classified incorrectly more than 90 times, that protein was called a candidate outlier protein (COP). To demonstrate the impact of COPs on the accuracy of classification models, we first identified them and removed them from the existing protein assemblage and trained the model with the residual proteins. For this purpose, SAAC and QSorder features have been used along with SVM, NB and KNN categories because they had the highest performance among the feature sets examined in this study. Figure 4 shows the results of 100 times tenfold CV after removing the COPs. As can be seen, the performance accuracy of the model has increased dramatically. Proteins removed through this method are listed in Additional file 5. Table 4 shows the percentage of moonlighting and non-MPs removed through the above methods. Column F.M. in this Table shows the number of proteins that were always misclassified. For example, the number 0.82 in the first cell row of this column indicates that 82% of the 64 proteins identified as COPs by the SAAC feature vector\using the SVM method perform 100 times tenfold CV were categorized erroneously F.M. For the two KNN models using the SAAC feature vector set and also the NB using the vector set the QSorder attribute shows the numbers 0.76 and 0.83, respectively. High F.M. shows that the classification of these proteins was very difficult by different classifications. Studies in recent years also confirm this, because none of the moonlighting and non-moonlighting protein classification methods that only used sequences have been able to achieve an accuracy higher than 0.77.

**Table 4:** Statistical information for outlier detection

Feature	Classifier	Moonlight	Non-MP	F.M*	Frequency
SAAC	SVM	0.35	0.65	0.82	64
SAAC	KNN	0.18	0.72	0.76	67
QSorder	NB	0.47	0.53	0.83	70

### Intersection of COPs

To obtain a more rigorous list, a combination of COP proteins of the top three methods was considered. Proteins are listed in Additional file 6. Identifying and examining the properties of these proteins can pave the way for more appropriate classification models. If a COP combination of the top 10 features and all classification models of this study are taken into consideration, proteins O75821 and P69786 will be found in10 feature vectors and P69797 in 9 feature vectors. Research shows that proteins P69786 and P69797 have been identified as non-MP proteins. One of the hypotheses that this study can make is that these two are moonlighting proteins. The reason is that classification models using different feature vectors tend to classify these two proteins as moonlighting proteins.

### Moonlighting candidates

We have obtained 13 proteins that have been identified as moonlighting through text mining but were available not in MoonProt database and were obtained from [1]. Eleven of the 13 proteins were identified by one of our model’s high-precision methods, the QSorder feature and the NB method. To increase the accuracy of the prediction, the method is repeated 100 times and the average probability is provided in Additional file 7. These proteins are most likely moonlighting. For example, Q944P7 protein, which is referred to as moonlighting in [35], in addition to peptidase activity, also has chaperone activity, which is independent of peptidase function.

List of moonlighting candidates sequence available in Additional file 8.

## Conclusion

MPs are important molecules in cell cycles. They have a significant role in regulatory activities and disease-related pathways. Experimental methods have their complications in detecting moonlighting proteins, therefore using computational methods has attracted much attention in detecting moonlighting proteins.

Many computational methods have been used to detect these proteins. However, studies that have used machine learning methods along with feature extraction are rare. In this study, 8 classification methods and 37 different feature vectors were used to detect moonlighting proteins. To evaluate the performance of the models, the proteins were divided into two parts: training (80%) and test (20%). Then, out of 37 feature vectors, 10 vectors were introduced that had higher performance than the others. Among the 10 superior feature vectors, SAAC vector using SVM and KNN methods and QSorder vector using NB method had the highest classification accuracy on the test data group. Another task in this study was to identify outlier proteins. To do this, NB with QSorder feature vector, SVM and KNN with SAAC feature vector were employed. In this method, tenfold cross validation has been performed 100 times on these models and at the time of execution, proteins that have been incorrectly classified as validation fold have been counted. If a protein was misaligned more than 90 times, that protein was known as a candidate outlier protein. The results show that outlier proteins can greatly reduce the accuracy of classifiers. Identification of these proteins and their properties can lead us to create more appropriate and accurate classification models, and this study can be the basis for future studies in this field. By studying non-MPs that were considered COPs, it is specified that their characteristics resemble MPs and it is better to drive them out of the non-MPs category, because it may later become clear that they were moonlighting proteins.

## Supplementary Information

**Additional file 1**. Moonlighting proteins sequence. List of moonlighting proteins sequence.

**Additional file 2**. Non-moonlighting proteins sequence. List of non-moonlighting proteins sequence.

**Additional file 3**. Description of feature vectors. Details of used ftrCool’s feature vector explained in this appendix.

**Additional file 4**. Performance evaluations. Performance evaluations 100 * 10 fold cross validation and test dataset for all models and feature vector.

**Additional file 5**. Detected outlier proteins. List of outlier proteins detected by three top models introduced in this appendix.

**Additional file 6**. Intersection cops. List of intersection outliers among the best models.

**Additional file 7**. Moonlighting candidates. List of moonlighting candidates.

**Additional file 8**. Moonlighting candidates sequence. List of moonlighting candidates sequence.

## Data Availability

All data generated or analyzed during this study are included in this published article and its supplementary information files. Data available in address https://github.com/karimrahimian/moonlight_proteins/tree/main/Data.

## Abbreviations

Acc: Accuracy
ADA: Ada boost
AUC: Area under curve
COP: Candidate outlier protein
DT: Decision tree
IF: Interest features
KNN: K-nearest-neighbor
LR: Logistic regression
MCC: Matthews correlation coefficient
MLP: Multi-layer perceptron
MPs: Moonlighting proteins
MS-WHIM: Molecular surface-weighted holistic invariant molecular
NB: Naive bayes
PPI: Protein–protein interaction
PseAAC: Pseudo-amino acid composition
PSSM: Position-specific scoring matrix
QSorder: Quasi sequence order
RF: Random forest
ROC: Receiver operating characteristic
SAAC: Splitted amino acid composition
SVM: Support vector machine
