# Correction to: Moonlighting protein prediction using physico‑chemical and evolutional properties via machine learning methods

**DOI:** 10.1186/s12859-021-04257-7

**Published:** 2021-07-09

**Authors:** Farshid Shirafkan, Sajjad Gharaghani, Karim Rahimian, Reza Hasan Sajedi, Javad Zahiri

**Affiliations:** 1 Laboratory of Bioinformatics and Drug Design, Institute of Biochemistry and Biophysics, University of Tehran, Tehran, Iran; 2 Bioinformatics and Computational Omics Lab (BioCOOL), Department of Biophysics, Faculty of Biological Sciences, Tarbiat Modares University, Tehran, Iran; 3 Department of Biochemistry, Faculty of Biological Sciences, Tarbiat Modares University, Tehran, Iran; 4 Department of Neuroscience, University of California San Diego, La Jolla, CA USA; 5 Department of Pediatrics, University of California San Diego, La Jolla, CA USA

## Correction to: BMC Bioinformatics (2021) 22:261 https://doi.org/10.1186/s12859-021-04194-5

Following the publication of the original article [1], the authors identified that Dr. Javad Zahiri was not assigned as the co-corresponding author in the published article.

The original article [1] has been corrected.
