# Supplementary material for: Moonlighting protein prediction using physico-chemical and evolutional properties via machine learning methods
Source: BMC Bioinformatics. 2021 May 24;22:261. doi: 10.1186/s12859-021-04194-5 (PMC8142502; doi:10.1186/s12859-021-04194-5)
Supplement: Supplementary file 3 — Additional file 3. Description of feature vectors. Details of used ftrCool’s feature vector explained in this appendix. [file 12859_2021_4194_MOESM3_ESM.docx]

**AAKpartComposition(**Grouped Amino Acid K Part Composition)

First, each protein sequence is divide into k equal groups, then the amino acid composition is calculated for each part. For each group, the length is obtained via the following formula:

$$L=\frac{l*(length of the sequence)}{k}$$

In some protein sequences, the last part can have a different length.[6]

**AAutoCor(**Amino Acid Autocorrelation-Auto covariance )

This feature build autocorrelation or autocovariance matrix or a combination of them. Moran, Greay, and NormalizeMBorto are autocorrelation methods and AC, ACC and CC are the autocovariance methods used by this feature vector.[6]

**CkSAApair(**Composition of k-Spaced Amino Acids pairs)

Ckssapair calculates the abundance of all amino acids with k (k can be set as 0,1,2,…,n) spaces. If k=0 then sequence fragment length is 2, if k=1 then sequence fragment length is 3, and so on. K-space amino acid pairs frequency is calculated by the following formula [6].

$$\frac{K-spaced}{Total length of sequence}$$

**CkSGAApair(Composition of k-Spaced Grouped Amino Acids pairs)**

G letter in this feature refer to group, so we can defined this feature as grouped k-space amino acids.First, each protein sequence is divide into groups, which can be defined by the user, then the CKSAApair calculate for each group [6].

**CTD (Composition_Transition_Distribution)**

Frequency of amino acids of a particular property is composition. For example if a sequence includes 10 type A residues and 16 type B residues, then the frequency of composition calculated as 10/(10+16) for A and 16/(10+16) for B. Transition (T) characterizes the percent frequency with which amino acids of a particular property is followed by amino acids of the other property. In the previous example with two type of amino acids transition characterize the percent frequency with which A is followed by B or b followed by A. Distribution of the given property of amino acid is described by five chain lengths, (first, 25%,50%,70%,100%). For more details, please check [2,6].

**CTDC (CTD Composition)**

**This feature is defined by thirteen properties, each property categorizes the amino acids into three group, then for each group composition is calculated** [6].

**CTDD** CTD Distribution

This feature is defined by fifteen values for each property. Check the references for more details about the values and property [6].

**DDE** Dipeptide Deviation from Expected Mean value

The DDE eigenvector is constructed by calculating three parameters: dipeptide composition (Dc), theoretical mean value (Tm), and theoretical variance (Tv). For more information about these three parameters please check [3,6].

**ExpectedValueAA** Expected Value for each Amino Acid

ExpectedvalueAA introduced by ftrCool package. It extracts k-mer in the sequence firstly, then compute expected values for each of them via the following formula:

$$ExpectedValue\left( k-mer \right)=\frac{frequency of k-mer}{(c_{1}*c_{2}\ldots*c_{k})}$$

where c_i is the number of codons that encrypt the i’th amino acid in the k-mer[6].

**ExpectedValueGAA** Expected Value for Grouped Amino Acid

This feature vector extended the ExpectedValueAA by defined amino acids groups. In this method, which introduced by ftrCool package for first time, amino acids could be splitted into user-defined groups, then, the for each group expected value of grouped is computed [6].

**ExpectedValueGKmerAA** (Expected Value for Grouped K-mer Amino Acid)

Similar to **ExpectedValueGAA and ExpectedValueAA** this feature vector is introduced by this package for the first time. In this method amino acids are first grouped into user-defined groups, then then expected value of grouped k-mer is calculated [6].

**ExpectedValueKmerAA** Expected Value for K-mer Amino Acid

This method calculates the expected value for each k-mer by dividing the frequency of kmer to multiplying frequency of each amino acid of the k-mer in the sequence [6].

**GrpDDE** Group Dipeptide Deviation from Expected Mean

In this method DDE is applied to user-defined groups of amino acids. ftrCool package introduce this model for the first time [6].

**SOCNumber** (Sequence Order Coupling Number)

Grantham and Schneider dissimilarity matrices used to calculate the dissimilarity between amino acid pairs. The distance between amino acid pairs is defined by d $\epsilon[1,nlag]$. SOCNumber method computes the sum of the dissimilarity of all amino acid pairs for each distance (d), so for each d there is a sum value, which indicate tau for d. Finally, the feature vector consists of tau values from both matrices. Thus, the length of feature vector is nlag*2 [6].

**kAAComposition** (k Amino Acid Composition)

**kAAComposition** calculates the frequency of each amino acid. The final feature vector consists of frequency (composition) of each amino acid [6].

**kGAAComposition** k Grouped Amino Acid Composition

In this method **kAAComposition** is applied to each user-defined grouped amino acid [6].

**PseKRAAC_(Ti)** Pseudo K_tuple Reduced Amino Acid Composition Type-i

There are 16 types of PseKRAAC method. In all method a grouping of amino acid (selecting by user) used to reduce the alphabetic amino acid. For more information about how to group amino acid please see [4]. Differences between 16 various types of pseKRAAC are displaying in table 1.

Table 1:16 various type of PseKRAAC functions

| PseKRAAC function | Description |
| --- | --- |
| PseKRAAC_T1 | PseKRAAC_type1(PseKRAAC_T1) contains Grp 2 to 20. |
| PseKRAAC_T2 | PseKRAAC_type2(PseKRAAC_T2) contains Grp 2-6,8,15,20. |
| PseKRAAC_T3A | 'PseKRAAC_T3A' contains Grp 2-20 |
| PseKRAAC_T3B | 'PseKRAAC_T3B' contains Grp 2-20. |
| PseKRAAC_T4 | PseKRAAC_type4(PseKRAAC_T4) contains Grp 5,8,9,11,13,20. |
| PseKRAAC_T5 | PseKRAAC_type5(PseKRAAC_T5) contains Grp 3,4,8,10,15,20. |
| PseKRAAC_T6A | PseKRAAC_T6A' contains Grp 4,5,20. |
| PseKRAAC_T6B | PseKRAAC_T6B' contains Grp 5. |
| PseKRAAC_T7 | PseKRAAC_type7(PseKRAAC_T7) contains Grp 2-20 |
| PseKRAAC_T8 | PseKRAAC_type8(PseKRAAC_T8) contains Grp 2-20 |
| PseKRAAC_T9 | PseKRAAC_type9(PseKRAAC_T9) contains Grp 2-20. |
| PseKRAAC_T10 | PseKRAAC_type10(PseKRAAC_T10) contains Grp 2-20 |
| PseKRAAC_T11 | PseKRAAC_type11(PseKRAAC_T11) contains Grp 2-20 |
| PseKRAAC_T12 | PseKRAAC_type12(PseKRAAC_T12) contains Grp 2-18,20 |
| PseKRAAC_T13 | PseKRAAC_type13(PseKRAAC_T13) contains Grp 4,12,17,20 |
| PseKRAAC_T14 | PseKRAAC_type14(PseKRAAC_T14) contains Grp 2-20. |
| PseKRAAC_T15 | PseKRAAC_type15(PseKRAAC_T15) contains Grp 2-16,20. |
| PseKRAAC_T16 | PseKRAAC_type16(PseKRAAC_T16) contains Grp 2-16,20. |

QSOrder Quasi Sequence Order

This function computes the quasi-sequence-order for sequences. First, the abundance of each amino acid is obtained and normalized by the following equation:

$$norm\left( A \right)=\frac{freq (A)}{sl+\frac{1}{w}\sum_{i=1}^{d} tau_{i}}$$

Which that freq(A) indicate frequency of desired amino acid, sl is the sequence length of amino acid, tau value is fiven by SOCnumber, d is a distance and W is windows length. For more details about Quasi Sequence order see [5,6]

SAAC Splitted Amino Acid Composition

In this method, the protein sequence spits into three parts. The first part is N-terminal, the third part is C-terminal and the middle part which contains all amino acids between N-terminal and C-terminal. The first and third parts are determined by the first numNterm and last numCterm amino acid respectively [6].

numNtem and numCterm could be defined by user and the default value is 25. Finally, kAAComposition is applied to compute composition of each of three parts.

SGAAC Splitted Group Amino Acid Composition

In this method **SAAC** is applied to each user-defined amino acid group [6].

**Reference**

[1] G. Govindan and A. S. Nair, "Composition, Transition and Distribution (CTD) — A dynamic feature for predictions based on hierarchical structure of cellular sorting," 2011 Annual IEEE India Conference, Hyderabad, India, 2011, pp. 1-6, doi: 10.1109/INDCON.2011.6139332.

[2] Dubchak I, Muchnik I, Holbrook SR, Kim SH. Prediction of protein folding class using global description of amino acid sequence. *Proc Natl Acad Sci U S A*. 1995;92(19):8700-8704. doi:10.1073/pnas.92.19.8700

[3] <https://www.frontiersin.org/articles/10.3389/fbioe.2020.584807/full>

[4] Chou, K.‐C. (2001), Prediction of protein cellular attributes using pseudo‐amino acid composition. Proteins, 43: 246-255. <https://doi.org/10.1002/prot.1035>

[5] Kuo-Chen Chou,Prediction of Protein Subcellular Locations by Incorporating Quasi-Sequence-Order Effect, Biochemical and Biophysical Research Communications,Volume 278, Issue 2,2000,Pages 477-483,ISSN 0006-291X, <https://doi.org/10.1006/bbrc.2000.3815>.

[6] ftrCool
