# Supplementary material for: Moonlighting protein prediction using physico-chemical and evolutional properties via machine learning methods
Source: BMC Bioinformatics. 2021 May 24;22:261. doi: 10.1186/s12859-021-04194-5 (PMC8142502; doi:10.1186/s12859-021-04194-5)
Supplement: Supplementary file 4 — Additional file 4. Performance evaluations. Performance evaluations 100 * 10 fold cross validation and test dataset for all models and feature vector. [file 12859_2021_4194_MOESM4_ESM.docx]

Table 1:Average* of accuracy of 100* 10 fold cross validation

|  | AdaBoost | KNN | NB | DT | LR | SVM | RF | MLP |
| --- | --- | --- | --- | --- | --- | --- | --- | --- |
| QSOrder | 0.724 | 0.738 | 0.745 | 0.659 | 0.739 | 0.745 | 0.739 | 0.715 |
| SAAC | 0.720 | 0.746 | 0.647 | 0.676 | 0.713 | 0.767 | 0.743 | 0.701 |
| IF | 0.703 | 0.680 | 0.726 | 0.669 | 0.703 | 0.725 | 0.728 | 0.690 |
| AAKpartComposition | 0.683 | 0.729 | 0.709 | 0.647 | 0.665 | 0.723 | 0.716 | 0.696 |
| ExpectedValueAA | 0.698 | 0.656 | 0.712 | 0.636 | 0.648 | 0.717 | 0.731 | 0.674 |
| DDE | 0.683 | 0.610 | 0.731 | 0.627 | 0.667 | 0.696 | 0.717 | 0.674 |
| GrpDDE | 0.698 | 0.651 | 0.652 | 0.640 | 0.696 | 0.694 | 0.697 | 0.664 |
| AAutoCor | 0.679 | 0.650 | 0.688 | 0.643 | 0.661 | 0.716 | 0.711 | 0.644 |
| PseKRAAC_T15 | 0.670 | 0.636 | 0.698 | 0.673 | 0.685 | 0.690 | 0.646 | 0.683 |
| CkSAApair | 0.673 | 0.646 | 0.671 | 0.616 | 0.660 | 0.720 | 0.708 | 0.678 |
| PseKRAAC_T7 | 0.701 | 0.690 | 0.538 | 0.669 | 0.687 | 0.710 | 0.700 | 0.669 |
| PseKRAAC_T6A | 0.685 | 0.640 | 0.532 | 0.688 | 0.699 | 0.709 | 0.707 | 0.689 |
| SGAAC | 0.676 | 0.653 | 0.672 | 0.658 | 0.616 | 0.707 | 0.690 | 0.670 |
| PseKRAAC_T2 | 0.672 | 0.615 | 0.691 | 0.665 | 0.687 | 0.688 | 0.630 | 0.687 |
| PseKRAAC_T1 | 0.681 | 0.615 | 0.685 | 0.666 | 0.688 | 0.684 | 0.633 | 0.672 |
| PseKRAAC_T16 | 0.682 | 0.616 | 0.679 | 0.666 | 0.690 | 0.685 | 0.640 | 0.671 |
| ExpectedValueGAA | 0.672 | 0.626 | 0.652 | 0.598 | 0.644 | 0.695 | 0.707 | 0.675 |
| PseKRAAC_T8 | 0.642 | 0.643 | 0.640 | 0.616 | 0.672 | 0.692 | 0.677 | 0.653 |
| kGAAComposition | 0.673 | 0.622 | 0.609 | 0.602 | 0.642 | 0.698 | 0.717 | 0.668 |
| PseKRAAC_T3B | 0.654 | 0.628 | 0.659 | 0.645 | 0.675 | 0.673 | 0.627 | 0.646 |
| PseKRAAC_T5 | 0.666 | 0.615 | 0.692 | 0.621 | 0.625 | 0.682 | 0.682 | 0.602 |
| CkSGAApair | 0.646 | 0.619 | 0.626 | 0.592 | 0.667 | 0.674 | 0.660 | 0.663 |
| PseKRAAC_T10 | 0.645 | 0.617 | 0.538 | 0.606 | 0.657 | 0.682 | 0.664 | 0.641 |
| PseKRAAC_T11 | 0.613 | 0.626 | 0.562 | 0.609 | 0.653 | 0.683 | 0.660 | 0.633 |
| kAAComposition | 0.608 | 0.581 | 0.699 | 0.617 | 0.606 | 0.621 | 0.680 | 0.586 |
| PseKRAAC_T12 | 0.632 | 0.606 | 0.441 | 0.631 | 0.634 | 0.679 | 0.672 | 0.625 |
| PseKRAAC_T6B | 0.618 | 0.614 | 0.507 | 0.596 | 0.604 | 0.637 | 0.622 | 0.610 |
| PseKRAAC_T3A | 0.621 | 0.551 | 0.615 | 0.609 | 0.627 | 0.613 | 0.544 | 0.611 |
| CTDC | 0.617 | 0.560 | 0.601 | 0.610 | 0.612 | 0.614 | 0.536 | 0.611 |
| CTD | 0.590 | 0.615 | 0.553 | 0.591 | 0.585 | 0.614 | 0.621 | 0.589 |
| PseKRAAC_T9 | 0.590 | 0.616 | 0.474 | 0.589 | 0.602 | 0.634 | 0.613 | 0.620 |
| CTDD | 0.601 | 0.615 | 0.517 | 0.586 | 0.598 | 0.619 | 0.618 | 0.577 |
| PseKRAAC_T14 | 0.598 | 0.545 | 0.612 | 0.588 | 0.613 | 0.604 | 0.566 | 0.603 |
| ExpectedValueKmerAA | 0.645 | 0.551 | 0.477 | 0.608 | 0.572 | 0.594 | 0.653 | 0.612 |
| SOCNumber | 0.596 | 0.554 | 0.599 | 0.593 | 0.613 | 0.614 | 0.518 | 0.613 |
| PseKRAAC_T13 | 0.601 | 0.617 | 0.460 | 0.596 | 0.578 | 0.609 | 0.616 | 0.563 |
| ExpectedValueGKmerAA | 0.589 | 0.566 | 0.472 | 0.597 | 0.600 | 0.614 | 0.613 | 0.555 |
| PseKRAAC_T4 | 0.577 | 0.613 | 0.457 | 0.585 | 0.595 | 0.603 | 0.597 | 0.576 |

* standard deviation for all method is less than 1.2.

Table 2: average* precision of 100* 10 fold cross validation

|  | AdaBoost | KNN | NB | DT | LR | SVM | RF | MLP |
| --- | --- | --- | --- | --- | --- | --- | --- | --- |
| QSOrder | 0.723 | 0.739 | 0.743 | 0.658 | 0.739 | 0.745 | 0.739 | 0.715 |
| SAAC | 0.720 | 0.746 | 0.647 | 0.676 | 0.713 | 0.767 | 0.743 | 0.701 |
| AAKpartComposition | 0.684 | 0.729 | 0.711 | 0.648 | 0.666 | 0.724 | 0.716 | 0.696 |
| ExpectedValueAA | 0.698 | 0.656 | 0.712 | 0.636 | 0.648 | 0.717 | 0.731 | 0.674 |
| DDE | 0.683 | 0.612 | 0.731 | 0.628 | 0.669 | 0.696 | 0.718 | 0.674 |
| AAutoCor | 0.680 | 0.653 | 0.689 | 0.643 | 0.661 | 0.716 | 0.711 | 0.644 |
| CkSAApair | 0.673 | 0.646 | 0.671 | 0.616 | 0.660 | 0.720 | 0.708 | 0.678 |
| PseKRAAC_T7 | 0.703 | 0.690 | 0.539 | 0.669 | 0.689 | 0.710 | 0.701 | 0.668 |
| PseKRAAC_T6A | 0.685 | 0.640 | 0.532 | 0.688 | 0.699 | 0.709 | 0.707 | 0.689 |
| SGAAC | 0.677 | 0.655 | 0.673 | 0.659 | 0.616 | 0.707 | 0.692 | 0.670 |
| PseKRAAC_T2 | 0.673 | 0.613 | 0.692 | 0.665 | 0.684 | 0.688 | 0.630 | 0.687 |
| PseKRAAC_T1 | 0.675 | 0.616 | 0.685 | 0.666 | 0.688 | 0.684 | 0.635 | 0.675 |
| PseKRAAC_T16 | 0.682 | 0.615 | 0.679 | 0.666 | 0.690 | 0.685 | 0.640 | 0.671 |
| ExpectedValueGAA | 0.673 | 0.626 | 0.652 | 0.598 | 0.645 | 0.695 | 0.707 | 0.675 |
| PseKRAAC_T8 | 0.642 | 0.643 | 0.645 | 0.618 | 0.679 | 0.699 | 0.678 | 0.655 |
| kGAAComposition | 0.673 | 0.622 | 0.609 | 0.602 | 0.642 | 0.698 | 0.717 | 0.668 |
| PseKRAAC_T3B | 0.655 | 0.629 | 0.655 | 0.644 | 0.673 | 0.673 | 0.627 | 0.646 |
| PseKRAAC_T5 | 0.661 | 0.611 | 0.694 | 0.622 | 0.626 | 0.682 | 0.682 | 0.602 |
| CkSGAApair | 0.646 | 0.619 | 0.626 | 0.592 | 0.668 | 0.674 | 0.660 | 0.663 |
| PseKRAAC_T10 | 0.645 | 0.616 | 0.537 | 0.606 | 0.656 | 0.682 | 0.663 | 0.641 |
| PseKRAAC_T11 | 0.612 | 0.626 | 0.561 | 0.609 | 0.652 | 0.682 | 0.659 | 0.632 |
| kAAComposition | 0.608 | 0.581 | 0.699 | 0.617 | 0.606 | 0.621 | 0.680 | 0.586 |
| PseKRAAC_T12 | 0.631 | 0.605 | 0.442 | 0.629 | 0.631 | 0.674 | 0.671 | 0.625 |
| PseKRAAC_T6B | 0.618 | 0.614 | 0.507 | 0.596 | 0.604 | 0.637 | 0.622 | 0.610 |
| PseKRAAC_T3A | 0.625 | 0.552 | 0.616 | 0.609 | 0.627 | 0.614 | 0.544 | 0.612 |
| CTDC | 0.617 | 0.562 | 0.605 | 0.612 | 0.611 | 0.615 | 0.536 | 0.612 |
| CTD | 0.592 | 0.613 | 0.555 | 0.590 | 0.581 | 0.612 | 0.621 | 0.589 |
| PseKRAAC_T9 | 0.587 | 0.614 | 0.472 | 0.581 | 0.6 | 0.632 | 0.610 | 0.617 |
| CTDD | 0.601 | 0.615 | 0.517 | 0.586 | 0.598 | 0.619 | 0.618 | 0.577 |
| PseKRAAC_T14 | 0.591 | 0.552 | 0.615 | 0.586 | 0.611 | 0.601 | 0.566 | 0.603 |
| ExpectedValueKmerAA | 0.645 | 0.551 | 0.471 | 0.608 | 0.572 | 0.593 | 0.653 | 0.612 |
| SOCNumber | 0.596 | 0.556 | 0.593 | 0.599 | 0.612 | 0.614 | 0.519 | 0.613 |
| PseKRAAC_T13 | 0.641 | 0.618 | 0.462 | 0.597 | 0.575 | 0.610 | 0.616 | 0.561 |
| ExpectedValueGKmerAA | 0.592 | 0.561 | 0.469 | 0.591 | 0.598 | 0.611 | 0.612 | 0.553 |
| PseKRAAC_T4 | 0.577 | 0.613 | 0.457 | 0.585 | 0.595 | 0.603 | 0.597 | 0.576 |
| IF | 0.7 | 0.681 | 0.739 | 0.678 | 0.701 | 0.721 | 0.721 | 0.690 |
| GrpDDE | 0.697 | 0.651 | 0.651 | 0.633 | 0.693 | 0.693 | 0.693 | 0.663 |
| PseKRAAC_T15 | 0.670 | 0.635 | 0.697 | 0.671 | 0.681 | 0.691 | 0.644 | 0.683 |

* standard deviation for all method is less than 1.2.

Table 3:average* Recall of 100 * 10 fold cross validation

|  | AdaBoost | KNN | NB | DT | LR | SVM | RF | MLP |
| --- | --- | --- | --- | --- | --- | --- | --- | --- |
| QSOrder | 0.724 | 0.738 | 0.744 | 0.659 | 0.739 | 0.745 | 0.739 | 0.715 |
| SAAC | 0.720 | 0.746 | 0.647 | 0.676 | 0.713 | 0.767 | 0.743 | 0.701 |
| IF | 0.703 | 0.680 | 0.726 | 0.669 | 0.703 | 0.725 | 0.728 | 0.690 |
| AAKpartComposition | 0.683 | 0.729 | 0.709 | 0.647 | 0.665 | 0.723 | 0.716 | 0.696 |
| ExpectedValueAA | 0.698 | 0.656 | 0.712 | 0.636 | 0.648 | 0.717 | 0.731 | 0.674 |
| DDE | 0.683 | 0.610 | 0.731 | 0.627 | 0.667 | 0.696 | 0.717 | 0.674 |
| GrpDDE | 0.698 | 0.651 | 0.652 | 0.640 | 0.696 | 0.694 | 0.697 | 0.664 |
| AAutoCor | 0.679 | 0.650 | 0.688 | 0.643 | 0.661 | 0.716 | 0.711 | 0.644 |
| PseKRAAC_T15 | 0.670 | 0.636 | 0.698 | 0.673 | 0.685 | 0.690 | 0.646 | 0.683 |
| CkSAApair | 0.673 | 0.646 | 0.671 | 0.616 | 0.660 | 0.720 | 0.708 | 0.678 |
| PseKRAAC_T7 | 0.701 | 0.690 | 0.538 | 0.669 | 0.687 | 0.710 | 0.700 | 0.669 |
| PseKRAAC_T6A | 0.685 | 0.640 | 0.532 | 0.688 | 0.699 | 0.709 | 0.707 | 0.689 |
| SGAAC | 0.676 | 0.653 | 0.672 | 0.658 | 0.616 | 0.707 | 0.690 | 0.670 |
| PseKRAAC_T2 | 0.672 | 0.615 | 0.691 | 0.665 | 0.687 | 0.688 | 0.630 | 0.687 |
| PseKRAAC_T1 | 0.675 | 0.616 | 0.685 | 0.666 | 0.688 | 0.684 | 0.635 | 0.675 |
| PseKRAAC_T16 | 0.682 | 0.616 | 0.679 | 0.666 | 0.690 | 0.685 | 0.640 | 0.671 |
| ExpectedValueGAA | 0.672 | 0.626 | 0.652 | 0.598 | 0.644 | 0.695 | 0.707 | 0.675 |
| PseKRAAC_T8 | 0.642 | 0.643 | 0.640 | 0.616 | 0.672 | 0.692 | 0.677 | 0.653 |
| kGAAComposition | 0.673 | 0.622 | 0.609 | 0.602 | 0.642 | 0.698 | 0.717 | 0.668 |
| PseKRAAC_T3B | 0.654 | 0.628 | 0.659 | 0.645 | 0.675 | 0.673 | 0.627 | 0.646 |
| PseKRAAC_T5 | 0.666 | 0.615 | 0.692 | 0.621 | 0.625 | 0.682 | 0.682 | 0.602 |
| CkSGAApair | 0.646 | 0.619 | 0.626 | 0.592 | 0.667 | 0.674 | 0.660 | 0.663 |
| PseKRAAC_T10 | 0.645 | 0.617 | 0.538 | 0.606 | 0.657 | 0.682 | 0.664 | 0.641 |
| PseKRAAC_T11 | 0.613 | 0.626 | 0.562 | 0.609 | 0.653 | 0.683 | 0.660 | 0.633 |
| kAAComposition | 0.608 | 0.581 | 0.699 | 0.617 | 0.606 | 0.621 | 0.680 | 0.586 |
| PseKRAAC_T12 | 0.632 | 0.606 | 0.441 | 0.631 | 0.634 | 0.679 | 0.672 | 0.625 |
| PseKRAAC_T6B | 0.618 | 0.614 | 0.507 | 0.596 | 0.604 | 0.637 | 0.622 | 0.610 |
| PseKRAAC_T3A | 0.621 | 0.551 | 0.615 | 0.609 | 0.627 | 0.613 | 0.544 | 0.611 |
| CTDC | 0.617 | 0.560 | 0.601 | 0.610 | 0.612 | 0.614 | 0.536 | 0.611 |
| CTD | 0.590 | 0.615 | 0.553 | 0.591 | 0.585 | 0.614 | 0.621 | 0.589 |
| PseKRAAC_T9 | 0.590 | 0.616 | 0.474 | 0.589 | 0.602 | 0.634 | 0.613 | 0.620 |
| CTDD | 0.601 | 0.615 | 0.517 | 0.586 | 0.598 | 0.619 | 0.618 | 0.577 |
| PseKRAAC_T14 | 0.598 | 0.545 | 0.612 | 0.588 | 0.613 | 0.604 | 0.566 | 0.603 |
| ExpectedValueKmerAA | 0.645 | 0.551 | 0.477 | 0.608 | 0.572 | 0.594 | 0.653 | 0.612 |
| SOCNumber | 0.596 | 0.554 | 0.599 | 0.593 | 0.613 | 0.614 | 0.518 | 0.613 |
| PseKRAAC_T13 | 0.601 | 0.617 | 0.460 | 0.596 | 0.578 | 0.609 | 0.616 | 0.563 |
| ExpectedValueGKmerAA | 0.589 | 0.566 | 0.472 | 0.597 | 0.600 | 0.614 | 0.613 | 0.555 |
| PseKRAAC_T4 | 0.577 | 0.613 | 0.457 | 0.585 | 0.595 | 0.603 | 0.597 | 0.576 |

* standard deviation for all method is less than 1.2.

Table 4:average* Auc of 100 * 10 fold cross validation

|  | AdaBoost | KNN | NB | DT | LR | SVM | RF | MLP |
| --- | --- | --- | --- | --- | --- | --- | --- | --- |
| QSOrder | 0.696 | 0.699 | 0.722 | 0.628 | 0.713 | 0.722 | 0.709 | 0.700 |
| SAAC | 0.694 | 0.712 | 0.633 | 0.642 | 0.692 | 0.748 | 0.711 | 0.686 |
| IF | 0.672 | 0.650 | 0.714 | 0.634 | 0.683 | 0.693 | 0.689 | 0.667 |
| AAKpartComposition | 0.653 | 0.689 | 0.689 | 0.608 | 0.642 | 0.701 | 0.677 | 0.683 |
| ExpectedValueAA | 0.669 | 0.638 | 0.704 | 0.608 | 0.631 | 0.677 | 0.675 | 0.657 |
| AAutoCor | 0.648 | 0.620 | 0.666 | 0.619 | 0.625 | 0.685 | 0.672 | 0.623 |
| CkSAApair | 0.642 | 0.617 | 0.672 | 0.586 | 0.644 | 0.686 | 0.647 | 0.662 |
| GrpDDE | 0.667 | 0.589 | 0.606 | 0.594 | 0.671 | 0.662 | 0.652 | 0.645 |
| DDE | 0.648 | 0.522 | 0.712 | 0.585 | 0.646 | 0.655 | 0.662 | 0.641 |
| PseKRAAC_T7 | 0.658 | 0.629 | 0.565 | 0.607 | 0.666 | 0.662 | 0.633 | 0.646 |
| PseKRAAC_T6A | 0.641 | 0.561 | 0.543 | 0.630 | 0.670 | 0.661 | 0.660 | 0.667 |
| ExpectedValueGAA | 0.642 | 0.594 | 0.649 | 0.553 | 0.633 | 0.649 | 0.644 | 0.659 |
| SGAAC | 0.636 | 0.582 | 0.641 | 0.613 | 0.567 | 0.669 | 0.647 | 0.650 |
| kGAAComposition | 0.640 | 0.588 | 0.592 | 0.566 | 0.629 | 0.652 | 0.656 | 0.654 |
| PseKRAAC_T15 | 0.603 | 0.597 | 0.633 | 0.605 | 0.615 | 0.621 | 0.599 | 0.618 |
| PseKRAAC_T2 | 0.608 | 0.573 | 0.632 | 0.600 | 0.631 | 0.624 | 0.581 | 0.629 |
| PseKRAAC_T1 | 0.611 | 0.583 | 0.629 | 0.603 | 0.632 | 0.623 | 0.582 | 0.625 |
| PseKRAAC_T16 | 0.621 | 0.583 | 0.613 | 0.604 | 0.630 | 0.623 | 0.583 | 0.616 |
| CkSGAApair | 0.606 | 0.582 | 0.600 | 0.552 | 0.640 | 0.633 | 0.606 | 0.646 |
| PseKRAAC_T8 | 0.591 | 0.548 | 0.627 | 0.546 | 0.640 | 0.643 | 0.624 | 0.624 |
| PseKRAAC_T3B | 0.597 | 0.576 | 0.612 | 0.576 | 0.616 | 0.618 | 0.599 | 0.601 |
| PseKRAAC_T5 | 0.614 | 0.526 | 0.653 | 0.549 | 0.601 | 0.636 | 0.616 | 0.561 |
| PseKRAAC_T10 | 0.594 | 0.529 | 0.561 | 0.541 | 0.627 | 0.634 | 0.606 | 0.615 |
| PseKRAAC_T11 | 0.563 | 0.537 | 0.568 | 0.537 | 0.622 | 0.631 | 0.600 | 0.602 |
| kAAComposition | 0.580 | 0.549 | 0.684 | 0.558 | 0.553 | 0.546 | 0.620 | 0.555 |
| PseKRAAC_T12 | 0.583 | 0.545 | 0.496 | 0.571 | 0.604 | 0.623 | 0.604 | 0.597 |
| PseKRAAC_T6B | 0.570 | 0.533 | 0.535 | 0.544 | 0.572 | 0.580 | 0.552 | 0.583 |
| CTDD | 0.551 | 0.568 | 0.562 | 0.541 | 0.536 | 0.535 | 0.565 | 0.547 |
| PseKRAAC_T9 | 0.544 | 0.535 | 0.490 | 0.547 | 0.572 | 0.579 | 0.544 | 0.589 |
| CTD | 0.537 | 0.566 | 0.565 | 0.547 | 0.516 | 0.535 | 0.565 | 0.556 |
| ExpectedValueKmerAA | 0.609 | 0.522 | 0.534 | 0.568 | 0.553 | 0.486 | 0.583 | 0.502 |
| PseKRAAC_T13 | 0.546 | 0.543 | 0.509 | 0.531 | 0.551 | 0.558 | 0.543 | 0.535 |
| PseKRAAC_T4 | 0.525 | 0.526 | 0.504 | 0.520 | 0.564 | 0.542 | 0.525 | 0.545 |
| ExpectedValueGKmerAA | 0.534 | 0.524 | 0.547 | 0.540 | 0.515 | 0.507 | 0.562 | 0.505 |
| PseKRAAC_T3A | 0.534 | 0.503 | 0.543 | 0.510 | 0.541 | 0.526 | 0.517 | 0.540 |
| PseKRAAC_T14 | 0.507 | 0.505 | 0.518 | 0.504 | 0.512 | 0.507 | 0.516 | 0.509 |
| CTDC | 0.520 | 0.511 | 0.501 | 0.512 | 0.500 | 0.503 | 0.503 | 0.499 |
| SOCNumber | 0.494 | 0.508 | 0.503 | 0.491 | 0.503 | 0.503 | 0.492 | 0.504 |

* standard deviation for all method is less than 1.2.

Table 5: average* F1 of 100 * 10 fold cross validation

|  | AdaBoost | KNN | NB | DT | LR | SVM | RF | MLP |
| --- | --- | --- | --- | --- | --- | --- | --- | --- |
| QSOrder | 0.691 | 0.697 | 0.716 | 0.616 | 0.708 | 0.717 | 0.705 | 0.691 |
| SAAC | 0.689 | 0.708 | 0.612 | 0.633 | 0.685 | 0.743 | 0.707 | 0.676 |
| IF | 0.666 | 0.644 | 0.706 | 0.625 | 0.676 | 0.690 | 0.686 | 0.660 |
| AAKpartComposition | 0.645 | 0.685 | 0.681 | 0.595 | 0.633 | 0.694 | 0.672 | 0.672 |
| ExpectedValueAA | 0.663 | 0.624 | 0.692 | 0.595 | 0.621 | 0.671 | 0.668 | 0.647 |
| AAutoCor | 0.640 | 0.611 | 0.658 | 0.608 | 0.616 | 0.680 | 0.665 | 0.613 |
| CkSAApair | 0.634 | 0.605 | 0.654 | 0.572 | 0.634 | 0.680 | 0.632 | 0.651 |
| GrpDDE | 0.659 | 0.564 | 0.594 | 0.580 | 0.663 | 0.655 | 0.643 | 0.635 |
| DDE | 0.642 | 0.462 | 0.705 | 0.573 | 0.638 | 0.648 | 0.654 | 0.634 |
| PseKRAAC_T7 | 0.652 | 0.618 | 0.514 | 0.591 | 0.657 | 0.655 | 0.615 | 0.635 |
| ExpectedValueGAA | 0.634 | 0.581 | 0.634 | 0.536 | 0.621 | 0.640 | 0.630 | 0.647 |
| PseKRAAC_T6A | 0.633 | 0.524 | 0.515 | 0.617 | 0.664 | 0.652 | 0.652 | 0.657 |
| SGAAC | 0.629 | 0.556 | 0.633 | 0.596 | 0.551 | 0.664 | 0.639 | 0.640 |
| kGAAComposition | 0.633 | 0.575 | 0.580 | 0.550 | 0.618 | 0.644 | 0.643 | 0.641 |
| CkSGAApair | 0.596 | 0.569 | 0.590 | 0.535 | 0.631 | 0.623 | 0.591 | 0.636 |
| PseKRAAC_T1 | 0.611 | 0.583 | 0.629 | 0.603 | 0.632 | 0.623 | 0.582 | 0.625 |
| PseKRAAC_T2 | 0.587 | 0.561 | 0.621 | 0.580 | 0.621 | 0.609 | 0.568 | 0.618 |
| PseKRAAC_T15 | 0.583 | 0.586 | 0.620 | 0.583 | 0.595 | 0.604 | 0.585 | 0.602 |
| PseKRAAC_T16 | 0.606 | 0.568 | 0.595 | 0.583 | 0.615 | 0.608 | 0.563 | 0.601 |
| PseKRAAC_T8 | 0.579 | 0.486 | 0.614 | 0.514 | 0.633 | 0.632 | 0.611 | 0.615 |
| PseKRAAC_T3B | 0.579 | 0.558 | 0.602 | 0.549 | 0.599 | 0.604 | 0.589 | 0.586 |
| PseKRAAC_T5 | 0.602 | 0.469 | 0.645 | 0.514 | 0.592 | 0.625 | 0.597 | 0.547 |
| PseKRAAC_T10 | 0.581 | 0.469 | 0.522 | 0.515 | 0.618 | 0.623 | 0.587 | 0.595 |
| kAAComposition | 0.569 | 0.537 | 0.674 | 0.541 | 0.532 | 0.505 | 0.601 | 0.538 |
| PseKRAAC_T11 | 0.547 | 0.481 | 0.543 | 0.503 | 0.614 | 0.620 | 0.582 | 0.585 |
| PseKRAAC_T12 | 0.567 | 0.517 | 0.419 | 0.548 | 0.594 | 0.607 | 0.580 | 0.581 |
| PseKRAAC_T6B | 0.554 | 0.488 | 0.496 | 0.522 | 0.562 | 0.559 | 0.517 | 0.571 |
| PseKRAAC_T9 | 0.526 | 0.487 | 0.458 | 0.526 | 0.562 | 0.560 | 0.508 | 0.578 |
| CTD | 0.518 | 0.550 | 0.534 | 0.524 | 0.480 | 0.489 | 0.543 | 0.536 |
| CTDD | 0.532 | 0.553 | 0.501 | 0.517 | 0.510 | 0.481 | 0.545 | 0.531 |
| PseKRAAC_T13 | 0.527 | 0.505 | 0.425 | 0.496 | 0.540 | 0.538 | 0.505 | 0.520 |
| ExpectedValueKmerAA | 0.599 | 0.510 | 0.462 | 0.552 | 0.540 | 0.376 | 0.555 | 0.389 |
| PseKRAAC_T4 | 0.505 | 0.468 | 0.444 | 0.485 | 0.552 | 0.514 | 0.482 | 0.533 |
| ExpectedValueGKmerAA | 0.512 | 0.509 | 0.441 | 0.507 | 0.452 | 0.406 | 0.543 | 0.461 |
| PseKRAAC_T3A | 0.480 | 0.484 | 0.471 | 0.425 | 0.487 | 0.471 | 0.506 | 0.505 |
| PseKRAAC_T14 | 0.435 | 0.488 | 0.444 | 0.444 | 0.423 | 0.426 | 0.494 | 0.422 |
| CTDC | 0.445 | 0.494 | 0.414 | 0.432 | 0.385 | 0.392 | 0.491 | 0.382 |
| SOCNumber | 0.398 | 0.489 | 0.426 | 0.399 | 0.393 | 0.389 | 0.480 | 0.392 |

* standard deviation for all method is less than 1.2.

Table 6: average* Matt of 100 * 10 fold cross validation

|  | AdaBoost | KNN | NB | DT | LR | SVM | RF | MLP |
| --- | --- | --- | --- | --- | --- | --- | --- | --- |
| AAKpartComposition | 0.317 | 0.414 | 0.381 | 0.235 | 0.288 | 0.410 | 0.384 | 0.365 |
| AAutoCor | 0.306 | 0.246 | 0.335 | 0.244 | 0.264 | 0.386 | 0.372 | 0.247 |
| CkSAApair | 0.295 | 0.246 | 0.335 | 0.176 | 0.287 | 0.395 | 0.365 | 0.324 |
| CkSGAApair | 0.224 | 0.173 | 0.203 | 0.114 | 0.286 | 0.289 | 0.245 | 0.290 |
| CTD | 0.085 | 0.145 | 0.132 | 0.100 | 0.045 | 0.102 | 0.151 | 0.123 |
| CTDC | 0.073 | 0.025 | 0.008 | 0.046 | 0.003 | 0.017 | 0.004 | -0.003 |
| CTDD | 0.114 | 0.149 | 0.133 | 0.085 | 0.091 | 0.108 | 0.150 | 0.097 |
| DDE | 0.311 | 0.066 | 0.428 | 0.183 | 0.296 | 0.339 | 0.388 | 0.296 |
| ExpectedValueAA | 0.352 | 0.284 | 0.403 | 0.225 | 0.263 | 0.387 | 0.422 | 0.315 |
| ExpectedValueGAA | 0.295 | 0.199 | 0.292 | 0.118 | 0.262 | 0.333 | 0.366 | 0.318 |
| ExpectedValueGKmerAA | 0.079 | 0.049 | 0.116 | 0.099 | 0.038 | 0.034 | 0.138 | 0.012 |
| ExpectedValueKmerAA | 0.230 | 0.043 | 0.074 | 0.145 | 0.105 | -0.056 | 0.221 | 0.008 |
| GrpDDE | 0.351 | 0.214 | 0.232 | 0.212 | 0.350 | 0.342 | 0.344 | 0.291 |
| IF | 0.361 | 0.311 | 0.427 | 0.287 | 0.371 | 0.407 | 0.412 | 0.340 |
| kAAComposition | 0.162 | 0.101 | 0.369 | 0.135 | 0.120 | 0.126 | 0.300 | 0.112 |
| kGAAComposition | 0.293 | 0.187 | 0.182 | 0.141 | 0.256 | 0.341 | 0.392 | 0.308 |
| PseKRAAC_T1 | 0.275 | 0.161 | 0.321 | 0.251 | 0.319 | 0.317 | 0.191 | 0.306 |
| PseKRAAC_T2 | 0.269 | 0.158 | 0.315 | 0.248 | 0.308 | 0.307 | 0.181 | 0.305 |
| PseKRAAC_T10 | 0.211 | 0.086 | 0.120 | 0.103 | 0.262 | 0.304 | 0.256 | 0.237 |
| PseKRAAC_T11 | 0.141 | 0.115 | 0.131 | 0.098 | 0.252 | 0.305 | 0.247 | 0.211 |
| PseKRAAC_T12 | 0.184 | 0.107 | -0.009 | 0.172 | 0.213 | 0.291 | 0.270 | 0.198 |
| PseKRAAC_T13 | 0.105 | 0.114 | 0.019 | 0.073 | 0.102 | 0.130 | 0.116 | 0.071 |
| PseKRAAC_T14 | 0.023 | 0.012 | 0.064 | 0.015 | 0.046 | 0.024 | 0.033 | 0.024 |
| PseKRAAC_T15 | 0.260 | 0.212 | 0.332 | 0.272 | 0.298 | 0.310 | 0.220 | 0.294 |
| PseKRAAC_T16 | 0.296 | 0.183 | 0.285 | 0.259 | 0.320 | 0.303 | 0.198 | 0.273 |
| PseKRAAC_T3A | 0.110 | 0.006 | 0.141 | 0.051 | 0.127 | 0.084 | 0.033 | 0.106 |
| PseKRAAC_T3B | 0.228 | 0.171 | 0.247 | 0.190 | 0.282 | 0.276 | 0.200 | 0.226 |
| PseKRAAC_T4 | 0.057 | 0.080 | 0.010 | 0.043 | 0.130 | 0.105 | 0.068 | 0.093 |
| PseKRAAC_T5 | 0.259 | 0.081 | 0.329 | 0.131 | 0.203 | 0.303 | 0.295 | 0.129 |
| PseKRAAC_T6A | 0.311 | 0.174 | 0.085 | 0.309 | 0.353 | 0.366 | 0.365 | 0.340 |
| PseKRAAC_T6B | 0.152 | 0.090 | 0.073 | 0.097 | 0.148 | 0.191 | 0.137 | 0.170 |
| PseKRAAC_T7 | 0.347 | 0.312 | 0.132 | 0.259 | 0.334 | 0.369 | 0.346 | 0.298 |
| PseKRAAC_T8 | 0.208 | 0.166 | 0.254 | 0.120 | 0.291 | 0.329 | 0.293 | 0.256 |
| PseKRAAC_T9 | 0.094 | 0.096 | -0.021 | 0.098 | 0.147 | 0.187 | 0.110 | 0.183 |
| QSOrder | 0.409 | 0.435 | 0.453 | 0.269 | 0.441 | 0.459 | 0.441 | 0.400 |
| SAAC | 0.400 | 0.451 | 0.276 | 0.299 | 0.390 | 0.505 | 0.450 | 0.373 |
| SGAAC | 0.294 | 0.211 | 0.292 | 0.256 | 0.149 | 0.364 | 0.324 | 0.303 |
| SOCNumber | -0.013 | 0.016 | 0.014 | -0.027 | 0.018 | 0.017 | -0.016 | 0.017 |

* standard deviation for all method is less than 1.2.

Table 7: average* Precision of test dataset

|  | AdaBoost | KNN | NB | DT | LR | SVM | RF | MLP |
| --- | --- | --- | --- | --- | --- | --- | --- | --- |
| QSOrder | 0.738 | 0.731 | 0.746 | 0.656 | 0.745 | 0.744 | 0.738 | 0.713 |
| SAAC | 0.721 | 0.748 | 0.663 | 0.681 | 0.707 | 0.753 | 0.744 | 0.705 |
| AAKpartComposition | 0.688 | 0.732 | 0.716 | 0.642 | 0.676 | 0.733 | 0.718 | 0.707 |
| IF | 0.705 | 0.676 | 0.711 | 0.676 | 0.700 | 0.714 | 0.724 | 0.683 |
| ExpectedValueAA | 0.705 | 0.667 | 0.714 | 0.636 | 0.643 | 0.719 | 0.731 | 0.678 |
| CkSAApair | 0.696 | 0.658 | 0.674 | 0.608 | 0.676 | 0.720 | 0.701 | 0.684 |
| AAutoCor | 0.679 | 0.661 | 0.697 | 0.644 | 0.658 | 0.719 | 0.704 | 0.649 |
| DDE | 0.691 | 0.623 | 0.728 | 0.620 | 0.661 | 0.696 | 0.704 | 0.672 |
| GrpDDE | 0.704 | 0.645 | 0.652 | 0.640 | 0.695 | 0.700 | 0.694 | 0.659 |
| PseKRAAC_T1 | 0.677 | 0.633 | 0.696 | 0.667 | 0.690 | 0.695 | 0.633 | 0.693 |
| PseKRAAC_T2 | 0.681 | 0.642 | 0.701 | 0.666 | 0.698 | 0.694 | 0.634 | 0.688 |
| PseKRAAC_T7 | 0.700 | 0.697 | 0.528 | 0.665 | 0.688 | 0.714 | 0.709 | 0.674 |
| PseKRAAC_T15 | 0.669 | 0.631 | 0.700 | 0.680 | 0.685 | 0.691 | 0.633 | 0.678 |
| PseKRAAC_T6A | 0.680 | 0.651 | 0.557 | 0.683 | 0.697 | 0.705 | 0.702 | 0.669 |
| PseKRAAC_T16 | 0.677 | 0.628 | 0.674 | 0.663 | 0.684 | 0.678 | 0.634 | 0.668 |
| SGAAC | 0.671 | 0.646 | 0.670 | 0.640 | 0.616 | 0.707 | 0.681 | 0.670 |
| PseKRAAC_T8 | 0.651 | 0.649 | 0.649 | 0.608 | 0.668 | 0.699 | 0.672 | 0.652 |
| PseKRAAC_T3B | 0.667 | 0.627 | 0.663 | 0.656 | 0.682 | 0.679 | 0.632 | 0.642 |
| ExpectedValueGAA | 0.668 | 0.622 | 0.659 | 0.615 | 0.618 | 0.694 | 0.704 | 0.662 |
| kGAAComposition | 0.682 | 0.616 | 0.598 | 0.598 | 0.618 | 0.691 | 0.711 | 0.665 |
| PseKRAAC_T5 | 0.671 | 0.610 | 0.706 | 0.608 | 0.607 | 0.680 | 0.680 | 0.575 |
| CkSGAApair | 0.630 | 0.621 | 0.636 | 0.584 | 0.671 | 0.678 | 0.653 | 0.658 |
| kAAComposition | 0.639 | 0.590 | 0.690 | 0.631 | 0.599 | 0.618 | 0.690 | 0.568 |
| PseKRAAC_T12 | 0.645 | 0.624 | 0.432 | 0.628 | 0.642 | 0.697 | 0.689 | 0.660 |
| PseKRAAC_T10 | 0.631 | 0.606 | 0.527 | 0.598 | 0.652 | 0.683 | 0.659 | 0.637 |
| PseKRAAC_T11 | 0.600 | 0.617 | 0.551 | 0.602 | 0.647 | 0.684 | 0.654 | 0.622 |
| CTDD | 0.606 | 0.631 | 0.528 | 0.605 | 0.600 | 0.633 | 0.636 | 0.576 |
| CTD | 0.598 | 0.624 | 0.564 | 0.599 | 0.581 | 0.629 | 0.633 | 0.577 |
| PseKRAAC_T9 | 0.599 | 0.612 | 0.483 | 0.599 | 0.604 | 0.651 | 0.608 | 0.629 |
| PseKRAAC_T3A | 0.624 | 0.559 | 0.598 | 0.607 | 0.625 | 0.607 | 0.548 | 0.611 |
| CTDC | 0.619 | 0.565 | 0.595 | 0.608 | 0.606 | 0.610 | 0.532 | 0.607 |
| PseKRAAC_T6B | 0.603 | 0.619 | 0.505 | 0.586 | 0.573 | 0.633 | 0.613 | 0.606 |
| SOCNumber | 0.599 | 0.572 | 0.595 | 0.600 | 0.606 | 0.612 | 0.540 | 0.610 |
| ExpectedValueKmerAA | 0.626 | 0.557 | 0.479 | 0.620 | 0.576 | 0.594 | 0.650 | 0.605 |
| PseKRAAC_T14 | 0.596 | 0.536 | 0.610 | 0.580 | 0.604 | 0.596 | 0.571 | 0.600 |
| PseKRAAC_T13 | 0.592 | 0.623 | 0.457 | 0.583 | 0.581 | 0.609 | 0.604 | 0.558 |
| PseKRAAC_T4 | 0.578 | 0.614 | 0.453 | 0.583 | 0.603 | 0.593 | 0.596 | 0.586 |
| ExpectedValueGKmerAA | 0.588 | 0.564 | 0.467 | 0.579 | 0.601 | 0.606 | 0.606 | 0.544 |

* standard deviation for all method is less than 1.5.

Table 8: average* accuracy of test dataset

|  | AdaBoost | KNN | NB | DT | LR | SVM | RF | MLP |
| --- | --- | --- | --- | --- | --- | --- | --- | --- |
| QSOrder | 0.738 | 0.731 | 0.746 | 0.656 | 0.745 | 0.744 | 0.738 | 0.713 |
| SAAC | 0.721 | 0.748 | 0.663 | 0.681 | 0.707 | 0.753 | 0.744 | 0.705 |
| AAKpartComposition | 0.688 | 0.732 | 0.716 | 0.642 | 0.676 | 0.733 | 0.718 | 0.707 |
| IF | 0.705 | 0.676 | 0.711 | 0.676 | 0.700 | 0.714 | 0.724 | 0.683 |
| ExpectedValueAA | 0.705 | 0.667 | 0.714 | 0.636 | 0.643 | 0.719 | 0.731 | 0.678 |
| CkSAApair | 0.696 | 0.658 | 0.674 | 0.608 | 0.676 | 0.720 | 0.701 | 0.684 |
| AAutoCor | 0.679 | 0.661 | 0.697 | 0.644 | 0.658 | 0.719 | 0.704 | 0.649 |
| DDE | 0.691 | 0.623 | 0.728 | 0.620 | 0.661 | 0.696 | 0.704 | 0.672 |
| GrpDDE | 0.704 | 0.645 | 0.652 | 0.640 | 0.695 | 0.700 | 0.694 | 0.659 |
| PseKRAAC_T1 | 0.677 | 0.633 | 0.696 | 0.667 | 0.690 | 0.695 | 0.633 | 0.693 |
| PseKRAAC_T2 | 0.688 | 0.648 | 0.688 | 0.666 | 0.687 | 0.701 | 0.634 | 0.694 |
| PseKRAAC_T7 | 0.700 | 0.697 | 0.528 | 0.665 | 0.688 | 0.714 | 0.709 | 0.674 |
| PseKRAAC_T15 | 0.669 | 0.631 | 0.700 | 0.680 | 0.685 | 0.691 | 0.633 | 0.678 |
| PseKRAAC_T6A | 0.680 | 0.651 | 0.557 | 0.683 | 0.697 | 0.705 | 0.702 | 0.669 |
| PseKRAAC_T16 | 0.677 | 0.628 | 0.674 | 0.663 | 0.684 | 0.678 | 0.634 | 0.668 |
| SGAAC | 0.671 | 0.646 | 0.670 | 0.640 | 0.616 | 0.707 | 0.681 | 0.670 |
| PseKRAAC_T8 | 0.651 | 0.649 | 0.649 | 0.608 | 0.668 | 0.699 | 0.672 | 0.652 |
| PseKRAAC_T3B | 0.667 | 0.627 | 0.663 | 0.656 | 0.682 | 0.679 | 0.632 | 0.642 |
| ExpectedValueGAA | 0.668 | 0.622 | 0.659 | 0.615 | 0.618 | 0.694 | 0.704 | 0.662 |
| kGAAComposition | 0.682 | 0.616 | 0.598 | 0.598 | 0.618 | 0.691 | 0.711 | 0.665 |
| PseKRAAC_T5 | 0.671 | 0.610 | 0.706 | 0.608 | 0.607 | 0.680 | 0.680 | 0.575 |
| CkSGAApair | 0.630 | 0.621 | 0.636 | 0.584 | 0.671 | 0.678 | 0.653 | 0.658 |
| kAAComposition | 0.639 | 0.590 | 0.690 | 0.631 | 0.599 | 0.618 | 0.690 | 0.568 |
| PseKRAAC_T12 | 0.645 | 0.624 | 0.432 | 0.628 | 0.642 | 0.697 | 0.689 | 0.660 |
| PseKRAAC_T10 | 0.631 | 0.606 | 0.527 | 0.598 | 0.652 | 0.683 | 0.659 | 0.637 |
| PseKRAAC_T11 | 0.600 | 0.617 | 0.551 | 0.602 | 0.647 | 0.684 | 0.654 | 0.622 |
| CTDD | 0.606 | 0.631 | 0.528 | 0.605 | 0.600 | 0.633 | 0.636 | 0.576 |
| CTD | 0.598 | 0.624 | 0.564 | 0.599 | 0.581 | 0.629 | 0.633 | 0.577 |
| PseKRAAC_T9 | 0.599 | 0.612 | 0.483 | 0.599 | 0.604 | 0.651 | 0.608 | 0.629 |
| PseKRAAC_T3A | 0.624 | 0.559 | 0.598 | 0.607 | 0.625 | 0.607 | 0.548 | 0.611 |
| CTDC | 0.619 | 0.565 | 0.595 | 0.608 | 0.606 | 0.610 | 0.532 | 0.607 |
| PseKRAAC_T6B | 0.603 | 0.619 | 0.505 | 0.586 | 0.573 | 0.633 | 0.613 | 0.606 |
| SOCNumber | 0.599 | 0.572 | 0.595 | 0.600 | 0.606 | 0.612 | 0.540 | 0.610 |
| ExpectedValueKmerAA | 0.626 | 0.557 | 0.479 | 0.620 | 0.576 | 0.594 | 0.650 | 0.605 |
| PseKRAAC_T14 | 0.596 | 0.536 | 0.610 | 0.580 | 0.604 | 0.596 | 0.571 | 0.600 |
| PseKRAAC_T13 | 0.592 | 0.623 | 0.457 | 0.583 | 0.581 | 0.609 | 0.604 | 0.558 |
| PseKRAAC_T4 | 0.578 | 0.614 | 0.453 | 0.583 | 0.603 | 0.593 | 0.596 | 0.586 |
| ExpectedValueGKmerAA | 0.588 | 0.564 | 0.467 | 0.579 | 0.601 | 0.606 | 0.606 | 0.544 |

* standard deviation for all method is less than 1.5.

Table 9: average* Auc of test dataset

|  | AdaBoost | KNN | NB | DT | LR | SVM | RF | MLP |
| --- | --- | --- | --- | --- | --- | --- | --- | --- |
| QSOrder | 0.711 | 0.696 | 0.726 | 0.621 | 0.718 | 0.723 | 0.708 | 0.700 |
| SAAC | 0.694 | 0.719 | 0.647 | 0.646 | 0.682 | 0.732 | 0.713 | 0.686 |
| AAKpartComposition | 0.660 | 0.695 | 0.699 | 0.605 | 0.653 | 0.714 | 0.681 | 0.694 |
| IF | 0.672 | 0.645 | 0.703 | 0.637 | 0.679 | 0.684 | 0.687 | 0.664 |
| ExpectedValueAA | 0.672 | 0.652 | 0.710 | 0.614 | 0.629 | 0.683 | 0.677 | 0.660 |
| CkSAApair | 0.664 | 0.627 | 0.673 | 0.581 | 0.664 | 0.686 | 0.640 | 0.670 |
| AAutoCor | 0.649 | 0.635 | 0.679 | 0.622 | 0.623 | 0.691 | 0.665 | 0.623 |
| PseKRAAC_T7 | 0.659 | 0.643 | 0.569 | 0.605 | 0.669 | 0.667 | 0.647 | 0.652 |
| GrpDDE | 0.673 | 0.586 | 0.608 | 0.595 | 0.668 | 0.669 | 0.654 | 0.642 |
| DDE | 0.655 | 0.542 | 0.710 | 0.580 | 0.642 | 0.659 | 0.651 | 0.641 |
| PseKRAAC_T6A | 0.638 | 0.579 | 0.558 | 0.624 | 0.675 | 0.662 | 0.658 | 0.654 |
| ExpectedValueGAA | 0.639 | 0.592 | 0.657 | 0.568 | 0.609 | 0.651 | 0.643 | 0.647 |
| SGAAC | 0.636 | 0.581 | 0.637 | 0.598 | 0.564 | 0.674 | 0.642 | 0.653 |
| PseKRAAC_T1 | 0.618 | 0.590 | 0.640 | 0.606 | 0.636 | 0.635 | 0.584 | 0.638 |
| kGAAComposition | 0.651 | 0.589 | 0.581 | 0.557 | 0.608 | 0.649 | 0.652 | 0.651 |
| PseKRAAC_T15 | 0.605 | 0.588 | 0.640 | 0.616 | 0.618 | 0.628 | 0.587 | 0.616 |
| PseKRAAC_T8 | 0.603 | 0.562 | 0.636 | 0.542 | 0.640 | 0.652 | 0.622 | 0.623 |
| PseKRAAC_T2 | 0.612 | 0.571 | 0.645 | 0.543 | 0.652 | 0.648 | 0.619 | 0.638 |
| CkSGAApair | 0.592 | 0.585 | 0.616 | 0.551 | 0.645 | 0.644 | 0.601 | 0.645 |
| PseKRAAC_T3B | 0.611 | 0.575 | 0.618 | 0.593 | 0.626 | 0.626 | 0.605 | 0.600 |
| PseKRAAC_T16 | 0.620 | 0.589 | 0.611 | 0.600 | 0.625 | 0.618 | 0.577 | 0.614 |
| PseKRAAC_T12 | 0.595 | 0.569 | 0.496 | 0.572 | 0.612 | 0.643 | 0.625 | 0.631 |
| PseKRAAC_T5 | 0.620 | 0.527 | 0.668 | 0.531 | 0.584 | 0.634 | 0.618 | 0.541 |
| kAAComposition | 0.616 | 0.558 | 0.674 | 0.580 | 0.541 | 0.542 | 0.636 | 0.537 |
| PseKRAAC_T10 | 0.583 | 0.523 | 0.551 | 0.533 | 0.625 | 0.637 | 0.600 | 0.609 |
| PseKRAAC_T11 | 0.550 | 0.533 | 0.553 | 0.533 | 0.618 | 0.635 | 0.595 | 0.585 |
| CTDD | 0.558 | 0.589 | 0.577 | 0.569 | 0.539 | 0.555 | 0.587 | 0.544 |
| CTD | 0.548 | 0.580 | 0.577 | 0.565 | 0.513 | 0.557 | 0.580 | 0.548 |
| PseKRAAC_T9 | 0.553 | 0.534 | 0.496 | 0.558 | 0.577 | 0.599 | 0.541 | 0.602 |
| PseKRAAC_T6B | 0.558 | 0.540 | 0.534 | 0.538 | 0.541 | 0.576 | 0.547 | 0.572 |
| ExpectedValueKmerAA | 0.590 | 0.523 | 0.536 | 0.582 | 0.553 | 0.490 | 0.580 | 0.499 |
| PseKRAAC_T13 | 0.539 | 0.554 | 0.518 | 0.526 | 0.551 | 0.560 | 0.534 | 0.531 |
| PseKRAAC_T4 | 0.531 | 0.530 | 0.506 | 0.523 | 0.573 | 0.535 | 0.524 | 0.556 |
| PseKRAAC_T3A | 0.541 | 0.519 | 0.545 | 0.509 | 0.545 | 0.525 | 0.522 | 0.543 |
| ExpectedValueGKmerAA | 0.532 | 0.523 | 0.541 | 0.520 | 0.513 | 0.503 | 0.553 | 0.494 |
| SOCNumber | 0.500 | 0.530 | 0.505 | 0.501 | 0.502 | 0.505 | 0.513 | 0.507 |
| PseKRAAC_T14 | 0.509 | 0.496 | 0.521 | 0.498 | 0.507 | 0.504 | 0.521 | 0.505 |
| CTDC | 0.526 | 0.515 | 0.500 | 0.512 | 0.500 | 0.504 | 0.501 | 0.500 |

* standard deviation for all method is less than 1.5.

Table 10:Average* Recall of test dataset

|  | AdaBoost | KNN | NB | DT | LR | SVM | RF | MLP |
| --- | --- | --- | --- | --- | --- | --- | --- | --- |
| QSOrder | 0.738 | 0.731 | 0.746 | 0.656 | 0.745 | 0.744 | 0.738 | 0.713 |
| SAAC | 0.721 | 0.748 | 0.663 | 0.681 | 0.707 | 0.753 | 0.744 | 0.705 |
| AAKpartComposition | 0.688 | 0.732 | 0.716 | 0.642 | 0.676 | 0.733 | 0.718 | 0.707 |
| IF | 0.705 | 0.676 | 0.711 | 0.676 | 0.700 | 0.714 | 0.724 | 0.683 |
| ExpectedValueAA | 0.705 | 0.667 | 0.714 | 0.636 | 0.643 | 0.719 | 0.731 | 0.678 |
| CkSAApair | 0.696 | 0.658 | 0.674 | 0.608 | 0.676 | 0.720 | 0.701 | 0.684 |
| AAutoCor | 0.679 | 0.661 | 0.697 | 0.644 | 0.658 | 0.719 | 0.704 | 0.649 |
| DDE | 0.691 | 0.623 | 0.728 | 0.620 | 0.661 | 0.696 | 0.704 | 0.672 |
| GrpDDE | 0.704 | 0.645 | 0.652 | 0.640 | 0.695 | 0.700 | 0.694 | 0.659 |
| PseKRAAC_T1 | 0.677 | 0.633 | 0.696 | 0.667 | 0.690 | 0.695 | 0.633 | 0.693 |
| PseKRAAC_T2 | 0.689 | 0.687 | 0.688 | 0.671 | 0.687 | 0.701 | 0.623 | 0.683 |
| PseKRAAC_T7 | 0.700 | 0.697 | 0.528 | 0.665 | 0.688 | 0.714 | 0.709 | 0.674 |
| PseKRAAC_T15 | 0.669 | 0.631 | 0.700 | 0.680 | 0.685 | 0.691 | 0.633 | 0.678 |
| PseKRAAC_T6A | 0.680 | 0.651 | 0.557 | 0.683 | 0.697 | 0.705 | 0.702 | 0.669 |
| PseKRAAC_T16 | 0.677 | 0.628 | 0.674 | 0.663 | 0.684 | 0.678 | 0.634 | 0.668 |
| SGAAC | 0.671 | 0.646 | 0.670 | 0.640 | 0.616 | 0.707 | 0.681 | 0.670 |
| PseKRAAC_T8 | 0.651 | 0.649 | 0.649 | 0.608 | 0.668 | 0.699 | 0.672 | 0.652 |
| PseKRAAC_T3B | 0.667 | 0.627 | 0.663 | 0.656 | 0.682 | 0.679 | 0.632 | 0.642 |
| ExpectedValueGAA | 0.668 | 0.622 | 0.659 | 0.615 | 0.618 | 0.694 | 0.704 | 0.662 |
| kGAAComposition | 0.682 | 0.616 | 0.598 | 0.598 | 0.618 | 0.691 | 0.711 | 0.665 |
| PseKRAAC_T5 | 0.671 | 0.610 | 0.706 | 0.608 | 0.607 | 0.680 | 0.680 | 0.575 |
| CkSGAApair | 0.630 | 0.621 | 0.636 | 0.584 | 0.671 | 0.678 | 0.653 | 0.658 |
| kAAComposition | 0.639 | 0.590 | 0.690 | 0.631 | 0.599 | 0.618 | 0.690 | 0.568 |
| PseKRAAC_T12 | 0.645 | 0.624 | 0.432 | 0.628 | 0.642 | 0.697 | 0.689 | 0.660 |
| PseKRAAC_T10 | 0.631 | 0.606 | 0.527 | 0.598 | 0.652 | 0.683 | 0.659 | 0.637 |
| PseKRAAC_T11 | 0.600 | 0.617 | 0.551 | 0.602 | 0.647 | 0.684 | 0.654 | 0.622 |
| CTDD | 0.606 | 0.631 | 0.528 | 0.605 | 0.600 | 0.633 | 0.636 | 0.576 |
| CTD | 0.598 | 0.624 | 0.564 | 0.599 | 0.581 | 0.629 | 0.633 | 0.577 |
| PseKRAAC_T9 | 0.599 | 0.612 | 0.483 | 0.599 | 0.604 | 0.651 | 0.608 | 0.629 |
| PseKRAAC_T3A | 0.624 | 0.559 | 0.598 | 0.607 | 0.625 | 0.607 | 0.548 | 0.611 |
| CTDC | 0.619 | 0.565 | 0.595 | 0.608 | 0.606 | 0.610 | 0.532 | 0.607 |
| PseKRAAC_T6B | 0.603 | 0.619 | 0.505 | 0.586 | 0.573 | 0.633 | 0.613 | 0.606 |
| SOCNumber | 0.599 | 0.572 | 0.595 | 0.600 | 0.606 | 0.612 | 0.540 | 0.610 |
| ExpectedValueKmerAA | 0.626 | 0.557 | 0.479 | 0.620 | 0.576 | 0.594 | 0.650 | 0.605 |
| PseKRAAC_T14 | 0.596 | 0.536 | 0.610 | 0.580 | 0.604 | 0.596 | 0.571 | 0.600 |
| PseKRAAC_T13 | 0.592 | 0.623 | 0.457 | 0.583 | 0.581 | 0.609 | 0.604 | 0.558 |
| PseKRAAC_T4 | 0.578 | 0.614 | 0.453 | 0.583 | 0.603 | 0.593 | 0.596 | 0.586 |
| ExpectedValueGKmerAA | 0.588 | 0.564 | 0.467 | 0.579 | 0.601 | 0.606 | 0.606 | 0.544 |

* standard deviation for all method is less than 1.5.

Table 11:Avergae * F1 of test dataset

|  | AdaBoost | KNN | NB | DT | LR | SVM | RF | MLP |
| --- | --- | --- | --- | --- | --- | --- | --- | --- |
| QSOrder | 0.714 | 0.699 | 0.727 | 0.617 | 0.721 | 0.723 | 0.711 | 0.697 |
| SAAC | 0.696 | 0.722 | 0.639 | 0.645 | 0.682 | 0.732 | 0.716 | 0.685 |
| AAKpartComposition | 0.660 | 0.697 | 0.698 | 0.597 | 0.652 | 0.713 | 0.683 | 0.691 |
| IF | 0.673 | 0.645 | 0.698 | 0.635 | 0.679 | 0.686 | 0.689 | 0.663 |
| ExpectedValueAA | 0.673 | 0.646 | 0.703 | 0.607 | 0.625 | 0.684 | 0.677 | 0.658 |
| AAutoCor | 0.648 | 0.633 | 0.677 | 0.616 | 0.620 | 0.692 | 0.665 | 0.621 |
| CkSAApair | 0.664 | 0.625 | 0.664 | 0.575 | 0.660 | 0.687 | 0.631 | 0.665 |
| GrpDDE | 0.674 | 0.570 | 0.603 | 0.588 | 0.668 | 0.669 | 0.650 | 0.639 |
| PseKRAAC_T7 | 0.660 | 0.638 | 0.514 | 0.594 | 0.667 | 0.668 | 0.639 | 0.650 |
| DDE | 0.655 | 0.496 | 0.709 | 0.573 | 0.640 | 0.659 | 0.647 | 0.641 |
| PseKRAAC_T6A | 0.636 | 0.550 | 0.545 | 0.615 | 0.674 | 0.661 | 0.656 | 0.649 |
| ExpectedValueGAA | 0.637 | 0.590 | 0.649 | 0.556 | 0.602 | 0.648 | 0.634 | 0.642 |
| SGAAC | 0.633 | 0.563 | 0.637 | 0.584 | 0.554 | 0.674 | 0.641 | 0.650 |
| PseKRAAC_T1 | 0.608 | 0.586 | 0.637 | 0.596 | 0.633 | 0.629 | 0.576 | 0.635 |
| PseKRAAC_T2 | 0.618 | 0.591 | 0.641 | 0.612 | 0.629 | 0.631 | 0.584 | 0.641 |
| kGAAComposition | 0.650 | 0.584 | 0.577 | 0.547 | 0.601 | 0.646 | 0.645 | 0.647 |
| CkSGAApair | 0.586 | 0.581 | 0.613 | 0.538 | 0.643 | 0.642 | 0.592 | 0.641 |
| PseKRAAC_T15 | 0.590 | 0.582 | 0.634 | 0.601 | 0.605 | 0.619 | 0.581 | 0.605 |
| PseKRAAC_T3B | 0.603 | 0.566 | 0.616 | 0.573 | 0.619 | 0.620 | 0.603 | 0.592 |
| PseKRAAC_T8 | 0.597 | 0.509 | 0.631 | 0.519 | 0.639 | 0.649 | 0.615 | 0.620 |
| PseKRAAC_T16 | 0.610 | 0.583 | 0.600 | 0.586 | 0.616 | 0.608 | 0.563 | 0.606 |
| kAAComposition | 0.614 | 0.553 | 0.671 | 0.571 | 0.525 | 0.505 | 0.631 | 0.529 |
| PseKRAAC_T5 | 0.613 | 0.478 | 0.669 | 0.491 | 0.580 | 0.630 | 0.606 | 0.529 |
| PseKRAAC_T12 | 0.588 | 0.551 | 0.407 | 0.553 | 0.608 | 0.638 | 0.611 | 0.624 |
| PseKRAAC_T10 | 0.575 | 0.471 | 0.521 | 0.504 | 0.622 | 0.633 | 0.586 | 0.599 |
| PseKRAAC_T11 | 0.538 | 0.480 | 0.538 | 0.503 | 0.614 | 0.630 | 0.582 | 0.565 |
| CTDD | 0.549 | 0.585 | 0.517 | 0.555 | 0.517 | 0.517 | 0.576 | 0.534 |
| PseKRAAC_T9 | 0.542 | 0.491 | 0.473 | 0.546 | 0.573 | 0.589 | 0.514 | 0.597 |
| CTD | 0.536 | 0.574 | 0.555 | 0.549 | 0.479 | 0.526 | 0.569 | 0.536 |
| PseKRAAC_T6B | 0.548 | 0.501 | 0.500 | 0.524 | 0.537 | 0.563 | 0.518 | 0.562 |
| PseKRAAC_T13 | 0.524 | 0.529 | 0.429 | 0.504 | 0.549 | 0.551 | 0.498 | 0.521 |
| PseKRAAC_T4 | 0.520 | 0.478 | 0.441 | 0.500 | 0.569 | 0.511 | 0.486 | 0.542 |
| ExpectedValueKmerAA | 0.587 | 0.519 | 0.468 | 0.574 | 0.549 | 0.377 | 0.556 | 0.386 |
| PseKRAAC_T3A | 0.495 | 0.510 | 0.474 | 0.421 | 0.503 | 0.476 | 0.519 | 0.512 |
| ExpectedValueGKmerAA | 0.515 | 0.514 | 0.435 | 0.494 | 0.449 | 0.398 | 0.541 | 0.452 |
| PseKRAAC_T14 | 0.445 | 0.486 | 0.453 | 0.436 | 0.419 | 0.430 | 0.508 | 0.420 |
| CTDC | 0.456 | 0.503 | 0.418 | 0.430 | 0.387 | 0.393 | 0.494 | 0.382 |
| SOCNumber | 0.405 | 0.522 | 0.436 | 0.407 | 0.392 | 0.390 | 0.508 | 0.400 |

* standard deviation for all method is less than 1.5.

Table 12:Average* Matt of test dataset

|  | AdaBoost | KNN | NB | DT | LR | SVM | RF | MLP |
| --- | --- | --- | --- | --- | --- | --- | --- | --- |
| QSOrder | 0.439 | 0.424 | 0.463 | 0.258 | 0.457 | 0.460 | 0.439 | 0.402 |
| SAAC | 0.402 | 0.463 | 0.303 | 0.311 | 0.377 | 0.477 | 0.455 | 0.379 |
| AAKpartComposition | 0.332 | 0.423 | 0.404 | 0.230 | 0.312 | 0.437 | 0.393 | 0.388 |
| IF | 0.363 | 0.301 | 0.404 | 0.300 | 0.364 | 0.384 | 0.403 | 0.330 |
| ExpectedValueAA | 0.364 | 0.308 | 0.415 | 0.235 | 0.259 | 0.398 | 0.427 | 0.326 |
| CkSAApair | 0.347 | 0.268 | 0.340 | 0.170 | 0.328 | 0.397 | 0.361 | 0.340 |
| PseKRAAC_T7 | 0.347 | 0.341 | 0.149 | 0.261 | 0.342 | 0.381 | 0.376 | 0.310 |
| AAutoCor | 0.312 | 0.277 | 0.362 | 0.252 | 0.262 | 0.398 | 0.363 | 0.254 |
| DDE | 0.331 | 0.134 | 0.427 | 0.176 | 0.287 | 0.343 | 0.361 | 0.294 |
| GrpDDE | 0.363 | 0.211 | 0.237 | 0.209 | 0.349 | 0.356 | 0.342 | 0.286 |
| PseKRAAC_T6A | 0.305 | 0.217 | 0.115 | 0.312 | 0.358 | 0.366 | 0.360 | 0.309 |
| PseKRAAC_T1 | 0.296 | 0.200 | 0.338 | 0.262 | 0.322 | 0.337 | 0.188 | 0.328 |
| PseKRAAC_T2 | 0.281 | 0.212 | 0.345 | 0.285 | 0.332 | 0.345 | 0.195 | 0.345 |
| PseKRAAC_T15 | 0.274 | 0.201 | 0.351 | 0.300 | 0.310 | 0.328 | 0.197 | 0.292 |
| ExpectedValueGAA | 0.289 | 0.194 | 0.308 | 0.149 | 0.214 | 0.340 | 0.364 | 0.295 |
| SGAAC | 0.291 | 0.207 | 0.288 | 0.231 | 0.150 | 0.371 | 0.308 | 0.307 |
| PseKRAAC_T16 | 0.293 | 0.197 | 0.283 | 0.258 | 0.313 | 0.294 | 0.187 | 0.269 |
| kGAAComposition | 0.318 | 0.185 | 0.161 | 0.125 | 0.212 | 0.334 | 0.384 | 0.301 |
| PseKRAAC_T8 | 0.233 | 0.228 | 0.273 | 0.110 | 0.291 | 0.347 | 0.282 | 0.254 |
| PseKRAAC_T3B | 0.268 | 0.173 | 0.262 | 0.240 | 0.305 | 0.301 | 0.218 | 0.223 |
| CkSGAApair | 0.199 | 0.180 | 0.234 | 0.105 | 0.301 | 0.311 | 0.236 | 0.290 |
| PseKRAAC_T12 | 0.217 | 0.163 | -0.012 | 0.181 | 0.235 | 0.343 | 0.331 | 0.273 |
| PseKRAAC_T5 | 0.280 | 0.084 | 0.365 | 0.107 | 0.171 | 0.307 | 0.303 | 0.086 |
| kAAComposition | 0.236 | 0.122 | 0.352 | 0.184 | 0.097 | 0.125 | 0.327 | 0.077 |
| PseKRAAC_T10 | 0.187 | 0.065 | 0.099 | 0.097 | 0.258 | 0.310 | 0.247 | 0.227 |
| PseKRAAC_T11 | 0.113 | 0.104 | 0.104 | 0.084 | 0.247 | 0.315 | 0.234 | 0.177 |
| CTDD | 0.130 | 0.193 | 0.165 | 0.140 | 0.099 | 0.168 | 0.199 | 0.094 |
| CTD | 0.108 | 0.175 | 0.153 | 0.130 | 0.036 | 0.163 | 0.189 | 0.109 |
| PseKRAAC_T9 | 0.114 | 0.095 | -0.010 | 0.122 | 0.158 | 0.229 | 0.109 | 0.208 |
| PseKRAAC_T6B | 0.130 | 0.120 | 0.069 | 0.081 | 0.084 | 0.183 | 0.122 | 0.149 |
| PseKRAAC_T3A | 0.143 | 0.039 | 0.147 | 0.060 | 0.142 | 0.083 | 0.044 | 0.118 |
| ExpectedValueKmerAA | 0.189 | 0.050 | 0.081 | 0.174 | 0.107 | -0.062 | 0.221 | -0.007 |
| PseKRAAC_T13 | 0.091 | 0.143 | 0.038 | 0.056 | 0.105 | 0.137 | 0.093 | 0.061 |
| PseKRAAC_T4 | 0.067 | 0.091 | 0.014 | 0.054 | 0.152 | 0.082 | 0.069 | 0.113 |
| ExpectedValueGKmerAA | 0.073 | 0.049 | 0.107 | 0.055 | 0.054 | 0.031 | 0.121 | -0.012 |
| SOCNumber | 0.022 | 0.066 | 0.032 | 0.020 | 0.020 | 0.044 | 0.027 | 0.046 |
| CTDC | 0.103 | 0.033 | 0.016 | 0.055 | 0.007 | 0.030 | 0.003 | 0.001 |
| PseKRAAC_T14 | 0.031 | -0.008 | 0.081 | -0.003 | 0.031 | 0.014 | 0.049 | 0.016 |

* standard deviation for all method is less than 1.5.
