# Supplementary material for: Moonlighting protein prediction using physico-chemical and evolutional properties via machine learning methods
Source: BMC Bioinformatics. 2021 May 24;22:261. doi: 10.1186/s12859-021-04194-5 (PMC8142502; doi:10.1186/s12859-021-04194-5)
Supplement: Supplementary file 5 — Additional file 5. Detected outlier proteins. List of outlier proteins detected by three top models introduced in this appendix. [file 12859_2021_4194_MOESM5_ESM.docx]

Table 1: Outliers detected by SVM using SAAC feature vector

| Non-MP Proteins | misclassify frequency | Moonlighting Proteins | misclassify frequency |
| --- | --- | --- | --- |
| sp\|P00550\|PTM3C_ECOLI | 100 | sp\|P19971\|TYPH_HUMAN | 96 |
| sp\|P03004\|DNAA_ECOLI | 100 | sp\|P15336\|ATF2_HUMAN | 100 |
| sp\|P04395\|3MG2_ECOLI | 100 | sp\|Q9H211\|CDT1_HUMAN | 100 |
| sp\|P0AFJ7\|PITA_ECOLI | 100 | sp\|P02730\|B3AT_HUMAN | 100 |
| sp\|P24177\|ACRD_ECOLI | 100 | sp\|P13569\|CFTR_HUMAN | 100 |
| sp\|P69797\|PTNAB_ECOLI | 100 | sp\|Q14152\|EIF3A_HUMAN | 100 |
| sp\|O14979\|HNRDL_HUMAN | 97 | sp\|Q04637\|IF4G1_HUMAN | 100 |
| sp\|O94811\|TPPP_HUMAN | 100 | sp\|P42566\|EPS15_HUMAN | 100 |
| sp\|P14866\|HNRPL_HUMAN | 100 | sp\|Q5BJF6\|ODFP2_HUMAN | 100 |
| sp\|P31943\|HNRH1_HUMAN | 100 | sp\|Q9H307\|PININ_HUMAN | 100 |
| sp\|P47813\|IF1AX_HUMAN | 100 | sp\|Q9CW03\|SMC3_MOUSE | 97 |
| sp\|Q13325\|IFIT5_HUMAN | 100 | sp\|P43274\|H14_MOUSE | 97 |
| sp\|Q15560\|TCEA2_HUMAN | 100 | sp\|P35846\|FOLR1_MOUSE | 100 |
| sp\|Q86WV1\|SKAP1_HUMAN | 100 | sp\|P42227\|STAT3_MOUSE | 100 |
| sp\|A2A5Z6\|SMUF2_MOUSE | 100 | sp\|P23202\|URE2_YEAST | 100 |
| sp\|Q5ISE2\|Z36L3_MOUSE | 94 | sp\|P33421\|SDH3_YEAST | 100 |
| sp\|Q62193\|RFA2_MOUSE | 92 | sp\|Q7JXV9\|VPS25_DROME | 92 |
| sp\|Q6Q473\|CLA4A_MOUSE | 99 | sp\|O74713\|HGT1_CANAX | 100 |
| sp\|Q8BIF2\|RFOX3_MOUSE | 100 | sp\|P03880\|ANI1_EMEND | 100 |
| sp\|Q8JZQ9\|EIF3B_MOUSE | 100 | sp\|P69786\|PTGCB_ECOLI | 100 |
| sp\|Q8JZX4\|SPF45_MOUSE | 100 | sp\|P9WQN9\|A85C_MYCTU | 97 |
| sp\|Q9ESD7\|DYSF_MOUSE | 97 | tr\|B9VJ59\|B9VJ59_9MONO | 100 |
| sp\|Q9WV06\|ANKR2_MOUSE | 100 | sp\|P43274\|H14_MOUSE | 100 |
| sp\|P06103\|EIF3B_YEAST | 100 | sp\|Q8WWY3\|PRP31_HUMAN | 100 |
| sp\|P11655\|SEC12_YEAST | 100 | sp\|O75821\|EIF3G_HUMAN | 100 |
| sp\|P25043\|PSB2_YEAST | 100 |  | |
| sp\|P25336\|MSH3_YEAST | 100 |  |  |
| sp\|P25582\|SPB1_YEAST | 100 |  |  |
| sp\|P32332\|OAC1_YEAST | 100 |  |  |
| sp\|P32368\|SAC1_YEAST | 100 |  |  |
| sp\|P33334\|PRP8_YEAST | 100 |  |  |
| sp\|P36160\|RPF2_YEAST | 100 |  |  |
| sp\|P38624\|PSB1_YEAST | 100 |  |  |
| sp\|P47176\|BCA2_YEAST | 100 |  |  |
| sp\|P53769\|CWC24_YEAST | 100 |  |  |
| sp\|Q04067\|EIF3G_YEAST | 100 |  |  |
| sp\|Q06106\|MRD1_YEAST | 100 |  |  |
| sp\|Q00539\|NAM8_YEAST | 93 |  |  |

Table 2: Outliers detected by NB using QSorder feature vector

| Non-MP proteins | misclassify frequency | Moonlighting Proteins | misclassify frequency |
| --- | --- | --- | --- |
| sp\|P03004\|DNAA_ECOLI | 100 | sp\|P19971\|TYPH_HUMAN | 100 |
| sp\|P04395\|3MG2_ECOLI | 98 | sp\|P15336\|ATF2_HUMAN | 100 |
| sp\|P0ABI4\|CORA_ECOLI | 98 | sp\|Q9H211\|CDT1_HUMAN | 100 |
| sp\|P69797\|PTNAB_ECOLI | 100 | sp\|Q8N3Y7\|RDHE2_HUMAN | 100 |
| sp\|O94811\|TPPP_HUMAN | 100 | sp\|P02730\|B3AT_HUMAN | 100 |
| sp\|P47813\|IF1AX_HUMAN | 100 | sp\|P13569\|CFTR_HUMAN | 100 |
| sp\|Q13257\|MD2L1_HUMAN | 100 | sp\|Q14152\|EIF3A_HUMAN | 100 |
| sp\|Q13325\|IFIT5_HUMAN | 100 | sp\|Q04637\|IF4G1_HUMAN | 100 |
| sp\|Q86WV1\|SKAP1_HUMAN | 95 | sp\|P42566\|EPS15_HUMAN | 100 |
| sp\|Q9H270\|VPS11_HUMAN | 100 | sp\|Q5BJF6\|ODFP2_HUMAN | 100 |
| sp\|Q9H492\|MLP3A_HUMAN | 100 | sp\|Q9H307\|PININ_HUMAN | 100 |
| sp\|Q9UJ71\|CLC4K_HUMAN | 100 | sp\|P43274\|H14_MOUSE | 96 |
| sp\|A2A5Z6\|SMUF2_MOUSE | 100 | sp\|P35846\|FOLR1_MOUSE | 100 |
| sp\|Q62193\|RFA2_MOUSE | 97 | sp\|P42227\|STAT3_MOUSE | 100 |
| sp\|Q6Q473\|CLA4A_MOUSE | 100 | sp\|P34960\|MMP12_MOUSE | 100 |
| sp\|Q8JZQ9\|EIF3B_MOUSE | 100 | sp\|P63159\|HMGB1_RAT | 93 |
| sp\|Q8JZX4\|SPF45_MOUSE | 100 | sp\|P17256\|KIME_RAT | 100 |
| sp\|Q8K273\|MMGT1_MOUSE | 100 | sp\|P08037\|B4GT1_BOVIN | 100 |
| sp\|Q9ESD7\|DYSF_MOUSE | 100 | sp\|P23202\|URE2_YEAST | 98 |
| sp\|Q9WV06\|ANKR2_MOUSE | 100 | sp\|P33421\|SDH3_YEAST | 100 |
| sp\|P06103\|EIF3B_YEAST | 100 | sp\|O74713\|HGT1_CANAX | 100 |
| sp\|P11655\|SEC12_YEAST | 100 | sp\|P03880\|ANI1_EMEND | 100 |
| sp\|P25043\|PSB2_YEAST | 100 | tr\|D5GCF2\|D5GCF2_TUBMM | 100 |
| sp\|P25336\|MSH3_YEAST | 100 | sp\|P05979\|PGH1_SHEEP | 100 |
| sp\|P25582\|SPB1_YEAST | 100 | sp\|P23872\|AES_ECOLI | 100 |
| sp\|P32332\|OAC1_YEAST | 98 | sp\|P69786\|PTGCB_ECOLI | 100 |
| sp\|P32368\|SAC1_YEAST | 100 | sp\|P07017\|MCP2_ECOLI | 100 |
| sp\|P33334\|PRP8_YEAST | 100 | sp\|P13299\|TEV1_BPT4 | 98 |
| sp\|P36160\|RPF2_YEAST | 100 | sp\|P9WQN9\|A85C_MYCTU | 100 |
| sp\|P38624\|PSB1_YEAST | 100 | sp\|Q2YVT4\|SLE1_STAAB | 100 |
| sp\|P47176\|BCA2_YEAST | 100 | tr\|Q9AIS0\|Q9AIS0_9STAP | 100 |
| sp\|P53769\|CWC24_YEAST | 99 | tr\|B9VJ59\|B9VJ59_9MONO | 100 |
| sp\|Q02486\|ABF2_YEAST | 100 | sp\|P43274\|H14_MOUSE | 98 |
| sp\|Q04067\|EIF3G_YEAST | 100 | sp\|Q8WWY3\|PRP31_HUMAN | 100 |
| sp\|Q06106\|MRD1_YEAST | 100 | sp\|O75821\|EIF3G_HUMAN | 100 |

Table 3: Outliers detected by KNN using SAAC feature vector

| Non-MP Proteins | misclassify frequency | Moonlighting Proteins | misclassify frequency |
| --- | --- | --- | --- |
| sp\|P00550\|PTM3C_ECOLI | 100 | sp\|Q00539\|NAM8_YEAST | 100 |
| sp\|P03004\|DNAA_ECOLI | 100 | sp\|P15336\|ATF2_HUMAN | 100 |
| sp\|P04395\|3MG2_ECOLI | 97 | sp\|P19338\|NUCL_HUMAN | 99 |
| sp\|P0ABI4\|CORA_ECOLI | 100 | sp\|P02730\|B3AT_HUMAN | 100 |
| sp\|P24177\|ACRD_ECOLI | 100 | sp\|Q14152\|EIF3A_HUMAN | 97 |
| sp\|P69797\|PTNAB_ECOLI | 100 | sp\|Q04637\|IF4G1_HUMAN | 100 |
| sp\|O14979\|HNRDL_HUMAN | 100 | sp\|P42566\|EPS15_HUMAN | 100 |
| sp\|O94811\|TPPP_HUMAN | 100 | sp\|Q5BJF6\|ODFP2_HUMAN | 100 |
| sp\|O95232\|LC7L3_HUMAN | 98 | sp\|Q9H307\|PININ_HUMAN | 100 |
| sp\|P04920\|B3A2_HUMAN | 100 | sp\|P45880\|VDAC2_HUMAN | 100 |
| sp\|P31943\|HNRH1_HUMAN | 100 | sp\|Q9CW03\|SMC3_MOUSE | 100 |
| sp\|P47813\|IF1AX_HUMAN | 100 | sp\|P35846\|FOLR1_MOUSE | 100 |
| sp\|Q13190\|STX5_HUMAN | 92 | sp\|P31230\|AIMP1_MOUSE | 99 |
| sp\|Q13257\|MD2L1_HUMAN | 96 | sp\|P23202\|URE2_YEAST | 97 |
| sp\|Q13325\|IFIT5_HUMAN | 100 | sp\|P33421\|SDH3_YEAST | 98 |
| sp\|Q15386\|UBE3C_HUMAN | 100 | sp\|O74713\|HGT1_CANAX | 100 |
| sp\|Q15560\|TCEA2_HUMAN | 95 | sp\|P03880\|ANI1_EMEND | 94 |
| sp\|Q86WV1\|SKAP1_HUMAN | 100 | sp\|P69786\|PTGCB_ECOLI | 100 |
| sp\|Q9H270\|VPS11_HUMAN | 97 | tr\|B9VJ59\|B9VJ59_9MONO | 95 |
| sp\|Q9H492\|MLP3A_HUMAN | 98 | sp\|O75821\|EIF3G_HUMAN | 100 |
| sp\|Q9HBJ8\|CLTRN_HUMAN | 100 | sp\|Q8WWY3\|PRP31_HUMAN | 100 |
| sp\|Q9UJ71\|CLC4K_HUMAN | 100 | sp\|P43274\|H14_MOUSE | 100 |
| sp\|A2A5Z6\|SMUF2_MOUSE | 100 |  | |
| sp\|Q5ISE2\|Z36L3_MOUSE | 100 |  |  |
| sp\|Q62193\|RFA2_MOUSE | 100 |  |  |
| sp\|Q6Q473\|CLA4A_MOUSE | 100 |  |  |
| sp\|Q7TPS5\|C2CD5_MOUSE | 100 |  |  |
| sp\|Q8JZQ9\|EIF3B_MOUSE | 100 |  |  |
| sp\|Q91Z92\|B3GT6_MOUSE | 100 |  |  |
| sp\|Q9ESD7\|DYSF_MOUSE | 100 |  |  |
| sp\|Q9R0Y1\|ASIC5_MOUSE | 99 |  |  |
| sp\|Q9WV06\|ANKR2_MOUSE | 100 |  |  |
| sp\|P06103\|EIF3B_YEAST | 100 |  |  |
| sp\|P25043\|PSB2_YEAST | 100 |  |  |
| sp\|P25336\|MSH3_YEAST | 100 |  |  |
| sp\|P25582\|SPB1_YEAST | 100 |  |  |
| sp\|P32332\|OAC1_YEAST | 100 |  |  |
| sp\|P32368\|SAC1_YEAST | 100 |  |  |
| sp\|P33334\|PRP8_YEAST | 100 |  |  |
| sp\|P36160\|RPF2_YEAST | 100 |  |  |
| sp\|P38624\|PSB1_YEAST | 100 |  |  |
| sp\|P47176\|BCA2_YEAST | 100 |  |  |
| sp\|Q02486\|ABF2_YEAST | 100 |  |  |
| sp\|Q04067\|EIF3G_YEAST | 100 |  |  |
| sp\|Q06106\|MRD1_YEAST | 100 |  |  |
