# Supplementary material for: Moonlighting protein prediction using physico-chemical and evolutional properties via machine learning methods
Source: BMC Bioinformatics. 2021 May 24;22:261. doi: 10.1186/s12859-021-04194-5 (PMC8142502; doi:10.1186/s12859-021-04194-5)
Supplement: Supplementary file 6 — Additional file 6. Intersection cops. List of intersection outliers among the best models. [file 12859_2021_4194_MOESM6_ESM.docx]

| Non_MP | | | | Moonlighting | | | |
| --- | --- | --- | --- | --- | --- | --- | --- |
| Row | ProteinID | Organism | Class | Row | ProteinID | Organism | Class |
| 1 | A2A5Z6 | SMUF2_MOUSE | Non_MP | **1** | O75821 | EIF3G_HUMAN | moonlight |
| 2 | Q13325 | IFIT5_HUMAN | Non_MP | **2** | Q8WWY3 | PRP31_HUMAN | moonlight |
| 3 | P47813 | IF1AX_HUMAN | Non_MP | **3** | P35846 | FOLR1_MOUSE | moonlight |
| 4 | Q9ESD7 | DYSF_MOUSE | Non_MP | **4** | P69786 | PTGCB_ECOLI | moonlight |
| 5 | P47176 | BCA2_YEAST | Non_MP | **5** | P42566 | EPS15_HUMAN | moonlight |
| 6 | P03004 | DNAA_ECOLI | Non_MP | **6** | O74713 | HGT1_CANAX | moonlight |
| 7 | P25336 | MSH3_YEAST | Non_MP | **7** | P15336 | ATF2_HUMAN | moonlight |
| 8 | P33334 | PRP8_YEAST | Non_MP | **8** | P43274 | H14_MOUSE | moonlight |
| 9 | Q06106 | MRD1_YEAST | Non_MP | **9** | Q04637 | IF4G1_HUMAN | moonlight |
| 10 | Q8JZQ9 | EIF3B_MOUSE | Non_MP | **10** | Q14152 | EIF3A_HUMAN | moonlight |
| 11 | P36160 | RPF2_YEAST | Non_MP | **11** | Q5BJF6 | ODFP2_HUMAN | moonlight |
| 12 | Q04067 | EIF3G_YEAST | Non_MP | **12** | P23202 | URE2_YEAST | moonlight |
| 13 | P38624 | PSB1_YEAST | Non_MP | **13** | P02730 | B3AT_HUMAN | moonlight |
| 14 | Q9WV06 | ANKR2_MOUSE | Non_MP | **14** | Q9H307 | PININ_HUMAN | moonlight |
| 15 | P06103 | EIF3B_YEAST | Non_MP | **15** | P03880 | ANI1_EMEND | moonlight |
| 16 | P32368 | SAC1_YEAST | Non_MP |  | | | |
| 17 | P69797 | PTNAB_ECOLI | Non_MP |  |  |  |  |
| 18 | O94811 | TPPP_HUMAN | Non_MP |  |  |  |  |
| 19 | P25582 | SPB1_YEAST | Non_MP |  |  |  |  |
| 20 | P25043 | PSB2_YEAST | Non_MP |  |  |  |  |
