# Supplementary material for: Moonlighting protein prediction using physico-chemical and evolutional properties via machine learning methods
Source: BMC Bioinformatics. 2021 May 24;22:261. doi: 10.1186/s12859-021-04194-5 (PMC8142502; doi:10.1186/s12859-021-04194-5)
Supplement: Supplementary file 7 — Additional file 7. Moonlighting candidates. List of moonlighting candidates. [file 12859_2021_4194_MOESM7_ESM.docx]

| **UniProtKB**  **ID** | **Moonlighting**  **Rate** | **Number of**  **Pfam Domains** | **Function 1** | **Function 2** |
| --- | --- | --- | --- | --- |
| Q9FI46 | 1 | 1 | Required for plastid  division, and involved in  cell differentiation and  regulation of the cell  division plane | Confers sensitivity to  cabbage leaf curl virus,  probably by hindering  its movement |
| B9DFA8 | 1 | 1 | Mitochondrial invertase  that cleaves sucrose into  glucose and fructose | Regulation of aerial tissue  development |
| O49606 | 1 | 1 | Stabilize and cross-link  actin filaments | Controls expression of  Flowering Locus C  gene via controlling  chromatin remodeling |
| P42744 | 1 | 1 | Regulatory subunit  ECR1-AXR1 E1 enzyme | Regulates the chromosomal  localization of meiotic  recombination by crossovers and subsequent synapsis,  probably through the activation of a CRL4 complex |
| P90992 | 1 | 1 | Transfer alpha  ketoglutarate across  inner mitochondrial  membrane | Control apoptosis through LIN-35/RB-like protein pathway |
| Q944P7 | 0.99 | 1 | Molecular chaperones | Leucine aminopeptidase  activity, role in insect defense |
| P17719 | 0.97 | 1 | Interact with vestigial, this  interaction may be  important for cell  proliferation and survival | Dihydrofolate reductase activity |
| Q18879 | 0.72 | 1 | Scaffolding protein within  caveolar membrane | uptake of lipids and proteins in intestinal cells |
| O44836 | 0.59 | 1 | embryogenesis/adult  development | pathogen resistance |
| Q94BN0 | 1 | 2 | component of the TAC1-mediated  telomerase activation pathway | mediating diverse hormone, stress, and metabolic responses |
| B5X582 | 1 | 2 | DNA helicase | DNA primase |
| G5EET6 | 0.97 | 2 | guanine nucleotide exchange factor for ARF6 | Limit microtubule growth  independent of arf-6,  inhibit axon regrowth |
| Q7KUT2 | 0.97 | 3 | ATP dependent serine protease | Chaperone function in assembly of inner membrane protein complexes |
